# Supplementary material for: Naturally acquired antibodies against 7 Streptococcus pneumoniae serotypes in Indigenous and non-Indigenous adults
Source: PLoS One. 2022 Apr 14;17(4):e0267051. doi: 10.1371/journal.pone.0267051 (PMC9009640; doi:10.1371/journal.pone.0267051)
Supplement: S1 Table — Fisher’s exact test compared proportions of Indigenous and non-Indigenous participants with IgG concentrations > 1.3 μg/ mL and > 1.0 μg/ mL for each serotype. (DOCX) [file pone.0267051.s001.docx]

|  | Participants with IgG pneumococcal capsular polysaccharide concentrations above 1.0 µg/ mL | | | Participants with IgG pneumococcal capsular polysaccharide concentrations above 1.3 µg/ mL | | |
| --- | --- | --- | --- | --- | --- | --- |
| Serotype | Indigenous  N (%) | Non-Indigenous  N (%) | Fisher’s exact test p- value | Indigenous  N (%) | Non-Indigenous  N (%) | Fisher’s exact test p- value |
| 3 | 9 (11.7) | 8 (12.5) | > 0.05 | 7 (9.1) | 3 (4.7) | > 0.05 |
| 6B | 57 (74.0) | 22 (34.4) | **< 0.0001** | 56 (72.7) | 18 (28.1) | **< 0.0001** |
| 9V | 55 (71.4) | 24 (37.5) | **< 0.0001** | 46 (59.7) | 18 (28.1) | **0.0002** |
| 14 | 72 (93.5) | 38 (59.4) | **< 0.0001** | 71 (92.2) | 35 (54.7) | **< 0.0001** |
| 19A | 62 (80.5) | 53 (82.8) | > 0.05 | 59 (76.6) | 45 (70.3) | > 0.05 |
| 19F | 54 (70.1) | 40 (62.5) | > 0.05 | 53 (68.8) | 37 (57.8) | > 0.05 |
| 23F | 33 (42.9) | 21 (32.8) | > 0.05 | 28 (36.4) | 18 (28.1) | > 0.05 |
