# Supplementary material for: Naturally acquired antibodies against 7 Streptococcus pneumoniae serotypes in Indigenous and non-Indigenous adults
Source: PLoS One. 2022 Apr 14;17(4):e0267051. doi: 10.1371/journal.pone.0267051 (PMC9009640; doi:10.1371/journal.pone.0267051)
Supplement: S2 Table — The goodness of the fit is reported by the r2 value; the p value determines if the slope is significantly non-zero. (DOCX) [file pone.0267051.s002.docx]

| Serotype | Non-Indigenous adults  r^2^, p-value | Indigenous adults  r^2^, p-value |
| --- | --- | --- |
| 3 | **0.07172, 0.0353** | 0.003944, > 0.05 |
| 6B | 0.003094, > 0.05 | 0.0005419, > 0.05 |
| 9V | 0.03104, > 0.05 | 0.001720, > 0.05 |
| 14 | **0.1166, 0.0071** | 0.0009479, > 0.05 |
| 19A | 0.01721, > 0.05 | 0.0008383, > 0.05 |
| 19F | 0.003314, > 0.05 | 0.0006019, > 0.05 |
| 23F | 0.004582, > 0.05 | 0.007891, > 0.05 |
