# Supplementary material for: Naturally acquired antibodies against 7 Streptococcus pneumoniae serotypes in Indigenous and non-Indigenous adults
Source: PLoS One. 2022 Apr 14;17(4):e0267051. doi: 10.1371/journal.pone.0267051 (PMC9009640; doi:10.1371/journal.pone.0267051)
Supplement: S3 Table — The goodness of the fit is reported by the r2 value; the p value determines if the slope is significantly non-zero. (DOCX) [file pone.0267051.s003.docx]

| Serotype | Non-Indigenous adults  r^2^, p-value | Indigenous adults  r^2^, p-value |
| --- | --- | --- |
| 3 | 0.01857, > 0.05 | 0.04447, > 0.05 |
| 6B | **0.1482, 0.0022** | 0.003236, > 0.05 |
| 9V | 0.04258, > 0.05 | 0.01333, > 0.05 |
| 14 | 0.02918, > 0.05 | 0.02345, > 0.05 |
| 19A | 0.01053, > 0.05 | 3.159e-5, > 0.05 |
| 19F | 0.009798, > 0.05 | 0.007549, > 0.05 |
| 23F | 0.007059, > 0.05 | 0.004430, > 0.05 |
