# Supplementary material for: Naturally acquired antibodies against 7 Streptococcus pneumoniae serotypes in Indigenous and non-Indigenous adults
Source: PLoS One. 2022 Apr 14;17(4):e0267051. doi: 10.1371/journal.pone.0267051 (PMC9009640; doi:10.1371/journal.pone.0267051)
Supplement: S4 Table — (DOCX) [file pone.0267051.s004.docx]

**Spearman correlation of IgM concentrations and age for non-Indigenous adults**

| Serotype | Spearman r, p-value |
| --- | --- |
| 3 | -0.04305, > 0.05 |
| 6B | -0.05264, > 0.05 |
| 9V | **-0.2793, 0.0307** |
| 14 | -0.08681, > 0.05 |
| 19A | -0.2157, > 0.05 |
| 19F | **-0.2547, 0.0495** |
| 23F | -0.1739, > 0.05 |
