# Supplementary material for: Naturally acquired antibodies against 7 Streptococcus pneumoniae serotypes in Indigenous and non-Indigenous adults
Source: PLoS One. 2022 Apr 14;17(4):e0267051. doi: 10.1371/journal.pone.0267051 (PMC9009640; doi:10.1371/journal.pone.0267051)
Supplement: S5 Table — Mann-Whitney U tests compared ages of males and females of the same ethnicity, there were no significant differences between groups. a One participant with an unknown age. (DOCX) [file pone.0267051.s005.docx]

|  |  | Age (years) | | |
| --- | --- | --- | --- | --- |
| Group | Number | Mean ± SEM | Median | Age Range |
| Indigenous Female | 38 | 38 (12.35) ^a^ | 37 | 19 – 64 |
| Indigenous Male | 37 | 43 (15.99) | 43 | 18 – 80 |
| Non-Indigenous Female | 42 | 44 (17.06) | 51 | 20 – 72 |
| Non-Indigenous Male | 21 | 48 (16.50) | 52 | 24 – 69 |
