# Supplementary material for: Naturally acquired antibodies against 7 Streptococcus pneumoniae serotypes in Indigenous and non-Indigenous adults
Source: PLoS One. 2022 Apr 14;17(4):e0267051. doi: 10.1371/journal.pone.0267051 (PMC9009640; doi:10.1371/journal.pone.0267051)
Supplement: S6 Table — Concentrations of antibodies were compared between males and females of the same ethnicity using a Mann-Whitney U test. (DOCX) [file pone.0267051.s006.docx]

| Antibody isotype | Serotype | Indigenous | | | Non-Indigenous | | |
| --- | --- | --- | --- | --- | --- | --- | --- |
|  |  | Female | Male | p value | Female | Male | p value |
| IgG | 3 | 0.39 (0.32 – 0.49) | 0.28 (0.21 – 0.38) | > 0.05 | 0.30 (0.23 – 0.40) | 0.30 (0.20 – 0.46) | > 0.05 |
|  | 6B | 1.63 (1.30 – 2.04) | 1.41(1.03 – 1.94) | > 0.05 | 0.49 (0.37 – 0.64) | 0.62 (0.34 – 1.13) | > 0.05 |
|  | 9V | 1.25 (1.10 – 1.43) | 1.04 (0.85 – 1.26) | > 0.05 | 0.62 (0.48 – 0.80) | 0.70 (0.5 – 0.98) | > 0.05 |
|  | 14 | 5.07 (3.91 – 6.56) | 2.90 (2.05 – 4.10) | 0.020 | 1.27 (0.95 – 1.69) | 1.51 (0.87 – 2.63) | > 0.05 |
|  | 19A | 2.65 (2.06 – 3.40) | 1.52 (2.06 – 3.40) | 0.0086 | 2.07 (1.12 – 2.04) | 1.44 (0.95 – 2.17) | > 0.05 |
|  | 19F | 1.99 (1.52 – 2.62) | 1.16 (0.85 – 1.58) | 0.0184 | 1.21 (0.90 – 1.62) | 1.18 (0.77 – 1.79) | > 0.05 |
|  | 23F | 0.70 (0.52 – 0.95) | 0.61(0.41 – 0.90) | > 0.05 | 0.58 (0.41 – 0.83) | 0.27 (0.15 – 0.49) | 0.041 |
| IgM | 3 | 0.39 (0.32 – 0.48) | 0.20 (0.16 – 0.25) | <0.0001 | 0.28 (0.23 – 0.34) | 0.19 (0.15 – 0.24) | 0.012 |
|  | 6B | 1.43(1.19 – 1.73) | 0.74 (0.57 – 0.96) | 0.0003 | 1.11 (0.90 – 1.37) | 0.71 (0.50 – 0.99) | > 0.05 |
|  | 9V | 0.48 (0.39 – 0.60) | 0.29 (0.22 – 0.38) | 0.0055 | 0.30 (0.23 – 0.39) | 0.17 (0.11 – 0.26) | 0.016 |
|  | 14 | 0.68 (0.55 – 0.83) | 0.47 (0.37 – 0.61) | > 0.05 | 0.53 (0.41 – 0.67) | 0.39 (0.30 – 0.52) | 0.041 |
|  | 19A | 1.86 (1.53 – 2.27) | 0.92 (0.73 – 1.18) | 0.0001 | 1.41 (1.13 – 1.76) | 0.82 (0.59 – 1.14) | 0.0066 |
|  | 19F | 1.60 (1.35 – 1.90) | 1.02 (0.80 – 1.31) | 0.0150 | 1.51 (1.21 – 1.88) | 0.91 (0.70 – 1.19) | 0.0037 |
|  | 23F | 0.29 (0.25 – 0.34) | 0.18 (0.14 – 0.23) | 0.0046 | 0.22 (0.18 – 0.26) | 0.15 (0.11 – 0.20) | 0.034 |
| IgA | 3 | 0.26 (0.20 – 0.34) | 0.19 (0.13 – 0.28) | > 0.05 | 0.17 (0.13 – 0.22) | 0.16 (0.12 – 0.22) | > 0.05 |
|  | 6B | 0.12 (0.10 – 0.14) | 0.12 (0.09 – 0.17) | > 0.05 | 0.09 (0.07 – 0.12) | 0.08 (0.05 – 0.11) | > 0.05 |
|  | 9V | 0.18 (0.14 – 0.23) | 0.22 (0.17 – 0.27) | > 0.05 | 0.09 (0.07 – 0.11) | 0.11 (0.09 – 0.14) | > 0.05 |
|  | 14 | 0.26 (0.20 – 0.33) | 0.21 (0.15 – 0.30) | > 0.05 | 0.12 (0.09 – 0.16) | 0.14 (0.09 – 0.22) | > 0.05 |
|  | 19A | 0.30 (0.22 – 0.40) | 0.24 (0.18 – 0.32) | > 0.05 | 0.15 (0.13 – 0.18) | 0.15 (0.11 – 0.20) | > 0.05 |
|  | 19F | 0.11 (0.08 – 0.14) | 0.11 (0.09 – 0.13) | > 0.05 | 0.08 (0.06 – 0.09) | 0.08 (0.06 – 0.11) | > 0.05 |
|  | 23F | 0.10 (0.08 – 0.14) | 0.11 (0.08 – 0.15) | > 0.05 | 0.06 (0.05 – 0.08) | 0.06 (0.04 – 0.08) | > 0.05 |
