# Supplementary material for: Naturally acquired antibodies against 7 Streptococcus pneumoniae serotypes in Indigenous and non-Indigenous adults
Source: PLoS One. 2022 Apr 14;17(4):e0267051. doi: 10.1371/journal.pone.0267051 (PMC9009640; doi:10.1371/journal.pone.0267051)
Supplement: S7 Table — (DOCX) [file pone.0267051.s007.docx]

| Age (years) | Characteristics | Country | Serotype | | | | | | | Reference |
| --- | --- | --- | --- | --- | --- | --- | --- | --- | --- | --- |
|  |  |  | 3 | 6B | 9V | 14 | 19A | 19F | 23F |  |
| 18 − 80 | Indigenous | Canada | 0.33 (0.28 – 0.40) | 1.49 (1.24 – 1.81) | 1.06 (0.95 – 1.18) | 3.06 (2.89 – 4.48) | 2.08 (1.69 – 2.55) | 1.49 (1.21 – 1.83) | 0.64 (0.50 – 0.81) | This study |
| 20 − 72 | non-Indigenous | Canada | 0.31 (0.25 – 0.38) | 0.52 (0.40 – 0.68) | 0.58 (0.48 – 0.70) | 1.32 (1.03 – 1.71) | 1.87 (1.47 – 2.38) | 1.21 (0.96 – 1.51) | 0.49 (0.36 – 0.67) | This study |
| 21-44 | General population | USA | 0.9 (0.6-1.3) | 0.7 (0.5-1.1) | 0.6 (0.4-0.9) | 1.8 (1.0-3.3) | 2.5 (1.6-4.0) | 1.6 (1.1-2.3) | 0.9 (0.5-1.5) | McFetridge et al, 2015 [1] |
| 20-45 | General population | USA | 0.8 (0.6-1.2) | 0.7 (0.4-1.1) | 0.7 (0.4-1.0) | 0.9 (0.5-1.7) | 1.9 (1.3-2.8) | 1.4 (0.9-2.3) | 0.5 (0.3-0.9) | McFetridge et al, 2015 [1] |
| 22-42 | General population | Finland | N/A | 2.1 (1.5-3.1) | 1.9 (1.3-2.8) | 1.2 (0.6-2.4) | N/A | 4.3 (2.6-6.9) | 1.8 (1.2-2.7) | Sankilampi et al. 1996 [2] |
| 30-64 | Women | Finland | ~ 0.6 | ~ 0.6 | ~ 0.5 | ~ 1.2 | N/A | N/A | ~ 0.9 | Simell et al. 2008 [3] |
| 30-64 | Men | Finland | ~ 0.55 | ~ 0.4 | ~ 0.52 | ~ 1.0 | N/A | N/A | ~ 0.7 | Simell et al. 2008 [3] |
| > 65 | Women | Finland | ~ 0.45 | ~ 0.45 | ~ 0.6 | ~ 1.5 | N/A | N/A | ~ 0.75 | Simell et al. 2008 [3] |
| > 65 | Men | Finland | ~ 0.3 | ~ 0.4 | ~ 0.7 | ~ 1.25 | N/A | N/A | ~ 0.75 | Simell et al. 2008 [3] |
| 55-74 | 62% of participants are Indigenous | USA | N/A | 1.89 (1.56-2.29) | N/A | 5.52 (4.50-6.76) | N/A | 3.57 (2.91-4.37) | N/A | Hammitt et al. 2011 [4] |
| 55-70 | Alaskan Indigenous | USA | N/A | 2.10 (0.88-5.02) | N/A | 2.61 (0.88-7.75) | N/A | 2.89 (1.62-5.13) | N/A | Miernyk et al. 2009 [5] |
| 55-70 | Alaskan Indigenous | USA | N/A | 7.54 (4.18-13.59) | N/A | 9.87 (4.79-20.37) | N/A | 9.58 (5.90-15.57) | N/A | Miernyk et al. 2009 [5] |
| 55-70 | Alaskan Indigenous | USA | N/A | 5.10 (2.63-9.991) | N/A | 19.69 (7.58-51.14) | N/A | 9.68 (5.90-15.89) | N/A | Miernyk et al. 2009 [5] |
| 55-70 | Alaskan Indigenous | USA | N/A | 1.35 (0.67-2.73) | N/A | 2.64 (1.13-6.15) | N/A | 2.18 (1.32-3.60) | N/A | Miernyk et al. 2009 [5] |
| 55-70 | Alaskan Indigenous | USA | N/A | 3.97 (1.57-10.05) | N/A | 3.82 (0.86-17.05) | N/A | 3.03 (1.66-5.53) | N/A | Miernyk et al. 2009 [5] |
| 55-70 | Alaskan Indigenous | USA | N/A | 2.94 (1.38-6.26) | N/A | 6.55 (3.20-13.40) | N/A | 3.78 (2.46-5.82) | N/A | Miernyk et al. 2009 [5] |
| 15-59 | Australian Indigenous | Australia | 0.66 (0.53-0.82) | 0.86 (0.65-1.13) | 0.78 (0.62-0.98) | 2.52 (1.69-3.76) | 7.31 (5.87-9.10) | 2.16 (1.70-2.75) | 0.84 (0.65-1.07) | Moberley et al. 2017 [6] |
| 15-59 | Australian non-Indigenous | Australia | 0.71 (0.47-1.08) | 0.42 (0.31-0.58) | 0.61 (0.44-0.86) | 1.00 (0.54-1.86) | 2.71 (1.94-3.79) | 0.94 (0.66-1.33) | 0.33 (0.22-0.51) | Moberley et al. 2017 [6] |
| 20-39 | General population | Netherlands | N/A | ~ 0.7 | ~ 0.42 | ~ 1.1 | N/A | ~ 4.25 | ~ 0.93 | Elberse et al. 2011 [7] |
| 40-59 | General population | Netherlands | N/A | ~ 0.6 | ~ 0.5 | ~ 1.6 | N/A | ~ 4.25 | ~ 0.91 | Elberse et al. 2011 [7] |
| 60-79 | General population | Netherlands | N/A | ~ 0.5 | ~ 0.45 | ~ 1.3 | N/A | ~ 3.4 | ~ 0.78 | Elberse et al. 2011 [7] |
| 50-70 | General population | UK | N/A | 0.42 (0.34-0.52) | 0.37 (0.30-0.45) | 1.49 (1.17-1.90) | N/A | 0.83 (0.69-1.00) | 0.43 (0.35-5.2) | Lazarus et al. 2011 [8] |
| 50-70 | General population | UK | N/A | 0.40 (0.29-0.55) | 0.31 (0.24-0.41) | 0.93 (0.64-1.35) | N/A | 0.68 (0.52-0.89) | 0.37 (0.28-0.50) | Lazarus et al. 2011 [8] |
| 50-64 | General population | USA | 1.0 (0.9-1.2) | 1.6 (1.4-2.0) | 1.3 (1.1-1.6) | 8.2 (7.1-9.4) | N/A | N/A | 1.6 (1.4-2.0) | Musher et al. 2010 [9] |
| 65-88 | General population | USA | 0.9 (0.7-1.1) | 1.5 (1.3-1.9) | 1.1 (0.9-1.3) | 7.4 (6.4-8.6) | N/A | N/A | 1.4 (1.2-1.7) | Musher et al. 2010 [9] |
| 20-66 | General population | USA | 1.3 (0.5, 2.8) | 2.9 (1.4, 8.2) | 2.0 (0.8, 5.6) | 2.2 (1.0, 6.6) | 4.7 (1.4, 9.8) | 3.2 (1.3, 10.7) | 7.3 (2.2, 18.6) | Park et al. 2021 [10] |

1. McFetridge R, Meulen AS, Folkerth SD, Hoekstra JA, Dallas M, Hoover PA, et al. Safety, tolerability, and immunogenicity of 15-valent pneumococcal conjugate vaccine in healthy adults. Vaccine. 2015;33(24):2793-9. Epub 2015/04/29. doi: 10.1016/j.vaccine.2015.04.025. PubMed PMID: 25913828.

2. Sankilampi U, Honkanen PO, Bloigu A, Herva E, Leinonen M. Antibody response to pneumococcal capsular polysaccharide vaccine in the elderly. J Infect Dis. 1996;173(2):387-93. Epub 1996/02/01. doi: 10.1093/infdis/173.2.387. PubMed PMID: 8568300.

3. Simell B, Lahdenkari M, Reunanen A, Kayhty H, Vakevainen M. Effects of ageing and gender on naturally acquired antibodies to pneumococcal capsular polysaccharides and virulence-associated proteins. Clin Vaccine Immunol. 2008;15(9):1391-7. Epub 2008/07/04. doi: 10.1128/CVI.00110-08. PubMed PMID: 18596205; PubMed Central PMCID: PMCPMC2546667.

4. Hammitt LL, Bulkow LR, Singleton RJ, Nuorti JP, Hummel KB, Miernyk KM, et al. Repeat revaccination with 23-valent pneumococcal polysaccharide vaccine among adults aged 55-74 years living in Alaska: no evidence of hyporesponsiveness. Vaccine. 2011;29(12):2287-95. Epub 2011/01/25. doi: 10.1016/j.vaccine.2011.01.029. PubMed PMID: 21255685.

5. Miernyk KM, Butler JC, Bulkow LR, Singleton RJ, Hennessy TW, Dentinger CM, et al. Immunogenicity and reactogenicity of pneumococcal polysaccharide and conjugate vaccines in alaska native adults 55-70 years of age. Clin Infect Dis. 2009;49(2):241-8. Epub 2009/06/16. doi: 10.1086/599824. PubMed PMID: 19522655.

6. Moberley S, Licciardi PV, Balloch A, Andrews R, Leach AJ, Kirkwood M, et al. Repeat pneumococcal polysaccharide vaccine in Indigenous Australian adults is associated with decreased immune responsiveness. Vaccine. 2017;35(22):2908-15. doi: 10.1016/j.vaccine.2017.04.040. PubMed PMID: 28455171.

7. Elberse KE, de Greeff SC, Wattimena N, Chew W, Schot CS, van de Pol JE, et al. Seroprevalence of IgG antibodies against 13 vaccine Streptococcus pneumoniae serotypes in the Netherlands. Vaccine. 2011;29(5):1029-35. Epub 2010/12/07. doi: 10.1016/j.vaccine.2010.11.054. PubMed PMID: 21129397.

8. Lazarus R, Clutterbuck E, Yu LM, Bowman J, Bateman EA, Diggle L, et al. A randomized study comparing combined pneumococcal conjugate and polysaccharide vaccination schedules in adults. Clin Infect Dis. 2011;52(6):736-42. doi: 10.1093/cid/cir003. PubMed PMID: 21367726.

9. Musher DM, Manof SB, Liss C, McFetridge RD, Marchese RD, Bushnell B, et al. Safety and antibody response, including antibody persistence for 5 years, after primary vaccination or revaccination with pneumococcal polysaccharide vaccine in middle-aged and older adults. J Infect Dis. 2010;201(4):516-24. Epub 2010/01/23. doi: 10.1086/649839. PubMed PMID: 20092407.

10. Park MA, Jenkins SM, Smith CY, Pyle RC, Sacco KA, Ryu E, et al. Pneumococcal serotype-specific cut-offs based on antibody responses to pneumococcal polysaccharide vaccination in healthy adults. Vaccine. 2021;39(21):2850-6. Epub 2021/04/27. doi: 10.1016/j.vaccine.2021.04.015. PubMed PMID: 33896666.
