# Supplementary material for: Naturally acquired antibodies against 7 Streptococcus pneumoniae serotypes in Indigenous and non-Indigenous adults
Source: PLoS One. 2022 Apr 14;17(4):e0267051. doi: 10.1371/journal.pone.0267051 (PMC9009640; doi:10.1371/journal.pone.0267051)
Supplement: S1 Appendix — Indigenous adults southern Ontario (Group 1), Indigenous adults northwestern Ontario (Group 2), non-Indigenous adults Thunder Bay (Group 3), and non-Indigenous adults Kenora (Group 4). Participant group, ID number, age and ethnicity are displayed. (DOCX) [file pone.0267051.s008.docx]

| Participant ID | Group | Age | Sex | Ethnicity |
| --- | --- | --- | --- | --- |
| ID001 | 1 | 50 | female | Indigenous |
| ID002 | 1 | 34 | male | Indigenous |
| ID003 | 1 | 22 | female | Indigenous |
| ID004 | 1 | 51 | female | Indigenous |
| ID005 | 1 | 57 | male | Indigenous |
| ID006 | 1 | 74 | male | Indigenous |
| ID007 | 1 | 60 | female | Indigenous |
| ID008 | 1 | 30 | female | Indigenous |
| ID009 | 1 | 29 | male | Indigenous |
| ID010 | 1 | 20 | female | Indigenous |
| ID011 | 1 | 54 | female | Indigenous |
| ID012 | 1 | 51 | female | Indigenous |
| ID013 | 1 | 55 | male | Indigenous |
| ID014 | 1 | 38 | female | Indigenous |
| ID015 | 1 | 34 | female | Indigenous |
| ID016 | 1 | 31 | female | Indigenous |
| ID017 | 1 | 53 | male | Indigenous |
| ID018 | 1 | 53 | male | Indigenous |
| ID019 | 1 | 34 | female | Indigenous |
| ID020 | 1 | 43 | male | Indigenous |
| ID021 | 1 | 54 | male | Indigenous |
| ID022 | 1 | 59 | female | Indigenous |
| ID023 | 1 | 58 | male | Indigenous |
| ID024 | 1 | 80 | male | Indigenous |
| ID025 | 1 | 53 | male | Indigenous |
| ID026 | 1 | 40 | male | Indigenous |
| ID027 | 1 | 46 | male | Indigenous |
| ID028 | 1 | unknown | female | Indigenous |
| ID029 | 1 | 59 | male | Indigenous |
| ID030 | 1 | 26 | female | Indigenous |
| ID031 | 2 | 21 | female | Indigenous |
| ID032 | 2 | 56 | female | Indigenous |
| ID033 | 2 | 40 | female | Indigenous |
| ID034 | 2 | 50 | male | Indigenous |
| ID035 | 2 | 54 | male | Indigenous |
| ID036 | 2 | 31 | male | Indigenous |
| ID037 | 2 | 30 | male | Indigenous |
| ID038 | 2 | 63 | male | Indigenous |
| ID039 | 2 | 54 | male | Indigenous |
| ID040 | 2 | 59 | male | Indigenous |
| ID041 | 2 | 31 | female | Indigenous |
| ID042 | 2 | 21 | male | Indigenous |
| ID043 | 2 | 20 | male | Indigenous |
| ID044 | 2 | 19 | female | Indigenous |
| ID045 | 2 | 26 | male | Indigenous |
| ID046 | 2 | 30 | male | Indigenous |
| ID047 | 2 | 32 | male | Indigenous |
| ID048 | 2 | 30 | female | Indigenous |
| ID049 | 2 | 31 | male | Indigenous |
| ID050 | 2 | 39 | female | Indigenous |
| ID051 | 2 | 24 | female | Indigenous |
| ID052 | 2 | 26 | female | Indigenous |
| ID053 | 2 | 38 | female | Indigenous |
| ID054 | 2 | 38 | male | Indigenous |
| ID055 | 2 | 37 | female | Indigenous |
| ID056 | 2 | 39 | male | Indigenous |
| ID057 | 2 | 64 | female | Indigenous |
| ID058 | 2 | 34 | male | Indigenous |
| ID059 | 2 | 31 | male | Indigenous |
| ID060 | 2 | 23 | female | Indigenous |
| ID061 | 2 | 42 | female | Indigenous |
| ID062 | 2 | 55 | male | Indigenous |
| ID063 | 2 | 27 | female | Indigenous |
| ID064 | 2 | 33 | female | Indigenous |
| ID065 | 2 | 50 | female | Indigenous |
| ID066 | 2 | 43 | female | Indigenous |
| ID067 | 2 | 22 | male | Indigenous |
| ID068 | 2 | 25 | male | Indigenous |
| ID069 | 2 | 23 | male | Indigenous |
| ID070 | 2 | 43 | female | Indigenous |
| ID071 | 2 | 24 | female | Indigenous |
| ID072 | 2 | 52 | male | Indigenous |
| ID073 | 2 | 46 | female | Indigenous |
| ID074 | 2 | 35 | female | Indigenous |
| ID075 | 2 | 18 | male | Indigenous |
| ID076 | 2 | 67 | male | Indigenous |
| ID077 | 2 | 41 | female | Indigenous |
| ID078 | 3 | 25 | female | non-Indigenous |
| ID079 | 3 | 22 | female | non-Indigenous |
| ID080 | 3 | 23 | female | non-Indigenous |
| ID081 | 3 | 25 | male | non-Indigenous |
| ID082 | 3 | 27 | male | non-Indigenous |
| ID083 | 3 | 24 | female | non-Indigenous |
| ID084 | 3 | 34 | female | non-Indigenous |
| ID085 | 3 | 27 | female | non-Indigenous |
| ID086 | 3 | 24 | male | non-Indigenous |
| ID087 | 3 | 23 | female | non-Indigenous |
| ID088 | 3 | 33 | female | non-Indigenous |
| ID089 | 3 | 24 | male | non-Indigenous |
| ID090 | 3 | 36 | male | non-Indigenous |
| ID091 | 3 | 26 | male | non-Indigenous |
| ID092 | 3 | 25 | female | non-Indigenous |
| ID093 | 3 | 24 | male | non-Indigenous |
| ID094 | 3 | 24 | female | non-Indigenous |
| ID095 | 3 | 49 | male | non-Indigenous |
| ID096 | 3 | 51 | female | non-Indigenous |
| ID097 | 3 | 52 | male | non-Indigenous |
| ID098 | 3 | 60 | male | non-Indigenous |
| ID099 | 3 | 59 | female | non-Indigenous |
| ID100 | 3 | 59 | female | non-Indigenous |
| ID101 | 3 | 55 | female | non-Indigenous |
| ID102 | 3 | 53 | male | non-Indigenous |
| ID103 | 3 | 54 | female | non-Indigenous |
| ID104 | 3 | 64 | female | non-Indigenous |
| ID105 | 3 | 55 | female | non-Indigenous |
| ID106 | 3 | 54 | male | non-Indigenous |
| ID107 | 3 | 54 | female | non-Indigenous |
| ID108 | 3 | 51 | female | non-Indigenous |
| ID109 | 3 | 59 | female | non-Indigenous |
| ID110 | 3 | 68 | male | non-Indigenous |
| ID111 | 3 | 61 | female | non-Indigenous |
| ID112 | 3 | 69 | male | non-Indigenous |
| ID113 | 3 | 51 | female | non-Indigenous |
| ID114 | 3 | 55 | male | non-Indigenous |
| ID115 | 3 | 60 | female | non-Indigenous |
| ID116 | 3 | 68 | male | non-Indigenous |
| ID117 | 3 | 68 | female | non-Indigenous |
| ID118 | 3 | 72 | female | non-Indigenous |
| ID119 | 3 | 62 | female | non-Indigenous |
| ID120 | 3 | 51 | male | non-Indigenous |
| ID121 | 3 | 62 | female | non-Indigenous |
| ID122 | 3 | 53 | female | non-Indigenous |
| ID123 | 4 | 45 | female | non-Indigenous |
| ID124 | 4 | 53 | female | non-Indigenous |
| ID125 | 4 | 22 | female | non-Indigenous |
| ID126 | 4 | 20 | female | non-Indigenous |
| ID127 | 4 | 52 | male | non-Indigenous |
| ID128 | 4 | 61 | female | non-Indigenous |
| ID129 | 4 | 25 | unknown | non-Indigenous |
| ID130 | 4 | 48 | female | non-Indigenous |
| ID131 | 4 | 57 | male | non-Indigenous |
| ID132 | 4 | 54 | female | non-Indigenous |
| ID133 | 4 | 27 | female | non-Indigenous |
| ID134 | 4 | 21 | female | non-Indigenous |
| ID135 | 4 | 23 | female | non-Indigenous |
| ID136 | 4 | 24 | female | non-Indigenous |
| ID137 | 4 | 27 | female | non-Indigenous |
| ID138 | 4 | 67 | male | non-Indigenous |
| ID139 | 4 | 60 | male | non-Indigenous |
| ID140 | 4 | 68 | female | non-Indigenous |
| ID141 | 4 | 57 | female | non-Indigenous |
