# Supplementary material for: Naturally acquired antibodies against 7 Streptococcus pneumoniae serotypes in Indigenous and non-Indigenous adults
Source: PLoS One. 2022 Apr 14;17(4):e0267051. doi: 10.1371/journal.pone.0267051 (PMC9009640; doi:10.1371/journal.pone.0267051)
Supplement: S2 Appendix — Indigenous adults southern Ontario (Group 1), Indigenous adults northwestern Ontario (Group 2), non-Indigenous adults Thunder Bay (Group 3), and non-Indigenous adults Kenora (Group 4). All data displayed are the original values. For our statistical analyses, the lower limit of detection was determined for each serotype according to the WHO pneumococcal ELISA protocol. The lower limits of detection are serotype 3 (0.100 μg/ mL), serotype 6B (0.167 μg/ mL), 9V (0.175 μg/ mL), 14 (0.339 μg/ mL), 19A (0.144 μg/ mL), 19F (0.166 μg/ mL) and 23F (0.144 μg/ mL). All values below the lower limits of detection were reported as half the value for statistical purposes. (DOCX) [file pone.0267051.s009.docx]

| Group | Participant ID | 3 | 6B | 9V | 14 | 19A | 19F | 23F |
| --- | --- | --- | --- | --- | --- | --- | --- | --- |
| 1 | ID001 | 0.2368 | 3.30386736 | 1.4645 | 11.6450 | 7.5008 | 8.2556 | 1.0875 |
| 1 | ID002 | 0.0433 | 0.3117 | 0.6715 | 0.4598 | 0.1569 | 0.1757 | 0.3565 |
| 1 | ID003 | 0.3129 | 2.4549 | 0.9733 | 8.0137 | 2.5717 | 2.6037 | 2.3370 |
| 1 | ID004 | 0.6083 | 4.4036 | 2.7033 | 13.5086 | 7.6915 | 3.3973 | 0.4849 |
| 1 | ID005 | 0.4903 | 2.8145 | 1.7915 | 13.7600 | 6.2188 | 9.7070 | 1.7841 |
| 1 | ID006 | 0.7878 | 1.5734 | 0.7606 | 3.0259 | 0.8602 | 0.4731 | 0.2532 |
| 1 | ID007 | 0.9769 | 3.7156 | 3.4148 | 66.9655 | 14.9613 | 7.1133 | 1.2422 |
| 1 | ID008 | 0.6314 | 2.5793 | 1.7804 | 45.6008 | 7.5774 | 5.5612 | 1.6946 |
| 1 | ID009 | 0.7131 | 6.2716 | 1.7423 | 8.3861 | 9.9067 | 5.6814 | 3.0386 |
| 1 | ID010 | 0.5408 | 0.6132 | 1.2014 | 13.1971 | 3.6406 | 4.6433 | 2.4796 |
| 1 | ID011 | 0.6717 | 1.5976 | 9.2182 | 45.6754 | 42.6086 | 37.9834 | 13.9481 |
| 1 | ID012 | 0.2163 | 1.1319 | 2.9370 | 20.6911 | 4.9059 | 4.2959 | 4.4085 |
| 1 | ID013 | 1.1585 | 7.7521 | 12.9554 | 58.3011 | 4.9143 | 9.1852 | 3.2021 |
| 1 | ID014 | 1.0497 | 0.9137 | 1.1027 | 5.4590 | 3.0224 | 2.6329 | 0.6331 |
| 1 | ID015 | 0.7427 | 1.7773 | 1.2381 | 14.7441 | 3.4033 | 4.0800 | 0.9097 |
| 1 | ID016 | 0.9631 | 3.2987 | 1.0376 | 8.4033 | 13.5694 | 6.7678 | 2.5187 |
| 1 | ID017 | 0.0728 | 7.1262 | 0.6198 | 3.4404 | 1.0535 | 0.4500 | 0.4222 |
| 1 | ID018 | 0.1359 | 0.3605 | 0.6853 | 2.0621 | 0.7802 | 0.7049 | 0.1093 |
| 1 | ID019 | 0.7559 | 1.5245 | 1.2042 | 7.8725 | 2.3374 | 3.1331 | 1.8300 |
| 1 | ID020 | 0.3745 | 2.7028 | 1.0245 | 3.7059 | 3.8451 | 1.5710 | 1.2932 |
| 1 | ID021 | 0.1389 | 0.8385 | 0.9560 | 2.5381 | 1.9520 | 1.4926 | 0.2663 |
| 1 | ID022 | 0.3170 | 2.1943 | 0.9824 | 1.3169 | 4.8069 | 1.6241 | 1.2658 |
| 1 | ID023 | 0.1474 | 0.6802 | 0.8845 | 1.7631 | 1.2510 | 1.2180 | 0.8168 |
| 1 | ID024 | 0.4759 | 3.0170 | 3.8559 | 14.9251 | 25.5290 | 15.9053 | 2.1036 |
| 1 | ID025 | 0.6358 | 2.9047 | 1.1344 | 2.2704 | 6.6951 | 3.1921 | 1.0923 |
| 1 | ID026 | 0.2454 | 0.5064 | 0.3160 | 1.8844 | 1.0308 | 0.6606 | 0.1378 |
| 1 | ID027 | 0.6119 | 4.3568 | 1.6379 | 3.9248 | 7.7488 | 3.5907 | 0.8549 |
| 1 | ID028 | 0.3071 | 0.5813 | 0.6003 | 5.3995 | 2.1307 | 1.3681 | 0.2349 |
| 1 | ID029 | 0.3548 | 0.5594 | 1.2404 | 1.0439 | 2.6478 | 1.7175 | 1.3369 |
| 1 | ID030 | 0.3278 | 0.4581 | 1.1075 | 1.4915 | 2.1318 | 1.3573 | 0.4105 |
| 2 | ID031 | 0.2337 | 2.5475 | 0.8399 | 2.9010 | 1.2204 | 0.6269 | 0.2681 |
| 2 | ID032 | 0.4066 | 1.2153 | 1.1451 | 9.4835 | 4.7107 | 1.2657 | 0.9970 |
| 2 | ID033 | 0.3358 | 1.3064 | 1.0433 | 2.9201 | 6.5794 | 1.9652 | 0.2628 |
| 2 | ID034 | 0.0658 | 0.3698 | 0.4536 | 0.7794 | 0.8023 | 0.3225 | 0.6403 |
| 2 | ID035 | 0.1592 | 1.2081 | 0.7146 | 1.9313 | 0.5932 | 0.6243 | 0.0881 |
| 2 | ID036 | 0.5116 | 3.2466 | 7.6089 | 9.3821 | 3.4873 | 2.8234 | 0.3638 |
| 2 | ID037 | 0.0672 | 3.3060 | 3.2709 | 1.8675 | 5.0980 | 0.4864 | 3.9304 |
| 2 | ID038 | 0.3743 | 1.3971 | 1.2362 | 23.1303 | 0.8214 | 0.7733 | 0.3162 |
| 2 | ID039 | 0.2917 | 0.5930 | 1.0384 | 2.0464 | 2.9246 | 2.6277 | 0.3304 |
| 2 | ID040 | 0.6711 | 2.4559 | 1.8923 | 3.3855 | 1.9115 | 1.2174 | 0.9991 |
| 2 | ID041 | 0.2122 | 2.5101 | 0.6894 | 2.4969 | 0.8658 | 0.5981 | 0.2749 |
| 2 | ID042 | 0.2652 | 3.2219 | 1.0208 | 1.1108 | 0.4253 | 0.4187 | 0.5213 |
| 2 | ID043 | 0.3933 | 0.2377 | 0.9113 | 0.6037 | 0.2898 | 0.2373 | 0.3615 |
| 2 | ID044 | 0.2337 | 4.1182 | 3.5466 | 22.3440 | 2.8988 | 1.5879 | 0.2426 |
| 2 | ID045 | 1.7179 | 1.1692 | 2.5243 | 2.3411 | 2.3905 | 1.0746 | 0.8880 |
| 2 | ID046 | 1.1285 | 1.7079 | 1.0135 | 13.3821 | 4.2906 | 2.5406 | 1.1790 |
| 2 | ID047 | 0.3514 | 1.1405 | 5.0861 | 2.2283 | 1.3352 | 1.5327 | 0.3421 |
| 2 | ID048 | 0.2895 | 2.1593 | 0.7472 | 6.3400 | 0.8555 | 1.5214 | 0.7200 |
| 2 | ID049 | 0.0265 | 0.3793 | 0.3347 | 0.5217 | 0.4564 | 0.2867 | 0.0546 |
| 2 | ID050 | 1.6118 | 7.1877 | 1.8524 | 4.3692 | 9.0775 | 5.4600 | 5.1198 |
| 2 | ID051 | 0.6409 | 1.7688 | 3.5319 | 6.6435 | 6.0074 | 3.1084 | 0.2579 |
| 2 | ID052 | 0.3775 | 0.6073 | 1.6246 | 5.2424 | 0.7564 | 0.5710 | 0.1378 |
| 2 | ID053 | 1.0327 | 0.5363 | 1.7879 | 4.7170 | 0.5321 | 0.3856 | 0.5656 |
| 2 | ID054 | 0.1394 | 3.6582 | 3.7658 | 3.6334 | 1.8972 | 1.0516 | 5.7036 |
| 2 | ID055 | 0.4856 | 1.0661 | 0.7610 | 3.6487 | 1.5828 | 0.6666 | 1.1852 |
| 2 | ID056 | 0.5704 | 25.7480 | 2.7229 | 4.6339 | 0.9447 | 0.7008 | 5.1666 |
| 2 | ID057 | 0.3014 | 0.8440 | 1.7279 | 36.8301 | 2.9044 | 6.2308 | 3.0471 |
| 2 | ID058 | 0.2407 | 1.6515 | 0.8032 | 10.9806 | 1.7200 | 0.7430 | 0.3879 |
| 2 | ID059 | 0.3411 | 1.4719 | 4.3109 | 4.1305 | 2.2190 | 1.1712 | 2.2166 |
| 2 | ID060 | 1.0530 | 2.2155 | 1.3238 | 2.0179 | 2.5750 | 2.0826 | 0.2602 |
| 2 | ID061 | 0.3617 | 1.0189 | 0.8560 | 6.1980 | 2.6802 | 1.9719 | 0.5504 |
| 2 | ID062 | 1.5311 | 12.7200 | 1.0841 | 10.2884 | 0.5011 | 2.1575 | 2.0972 |
| 2 | ID063 | 0.5622 | 4.6150 | 4.4501 | 5.8471 | 5.7446 | 4.6655 | 2.4799 |
| 2 | ID064 | 0.6041 | 3.2847 | 1.1118 | 3.5328 | 3.1650 | 1.5417 | 0.3815 |
| 2 | ID065 | 0.6493 | 1.5273 | 1.2371 | 5.6625 | 1.1913 | 0.6493 | 1.6391 |
| 2 | ID066 | 0.1500 | 0.8396 | 1.0682 | 1.6401 | 1.4219 | 0.8921 | 0.7760 |
| 2 | ID067 | 0.1416 | 0.4195 | 1.5296 | 0.3150 | 1.0869 | 1.6075 | 0.1413 |
| 2 | ID068 | 0.2997 | 1.3685 | 1.3374 | 3.7122 | 3.3901 | 4.8468 | 1.1561 |
| 2 | ID069 | 0.4860 | 2.1668 | 0.9289 | 11.9220 | 2.5367 | 2.7239 | 6.0620 |
| 2 | ID070 | 0.0999 | 15.0824 | 1.1064 | 5.1353 | 1.3714 | 1.3491 | 0.9672 |
| 2 | ID071 | 0.2052 | 16.2548 | 1.7479 | 4.4213 | 1.6423 | 0.9393 | 1.5041 |
| 2 | ID072 | 1.0819 | 0.6458 | 0.4253 | 2.7989 | 1.2620 | 0.9317 | 0.7691 |
| 2 | ID073 | 0.5399 | 3.6007 | 1.5403 | 3.0374 | 4.1852 | 2.6648 | 0.5006 |
| 2 | ID074 | 0.1441 | 1.5487 | 2.0036 | 1.6638 | 1.4413 | 1.1337 | 0.3628 |
| 2 | ID075 | 0.2684 | 3.3230 | 1.7986 | 7.8846 | 2.3625 | 4.7439 | 1.6593 |
| 2 | ID076 | 0.3150 | 4.4424 | 1.0527 | 4.3871 | 1.8730 | 3.5538 | 1.1378 |
| 2 | ID077 | 0.1735 | 1.3611 | 1.7787 | 2.2945 | 2.4960 | 1.3434 | 0.3587 |
| 3 | ID078 | 1.3043 | 3.0949 | 0.6559 | 4.9771 | 5.0367 | 8.7570 | 1.7651 |
| 3 | ID079 | 1.1281 | 0.8067 | 1.7626 | 2.8890 | 4.0822 | 2.8431 | 2.3306 |
| 3 | ID080 | 0.5621 | 0.6495 | 0.4716 | 0.4358 | 1.4842 | 1.3634 | 0.2745 |
| 3 | ID081 | 0.2441 | 0.6799 | 0.2933 | 0.1701 | 1.0948 | 0.7620 | 0.1237 |
| 3 | ID082 | 0.6885 | 4.5989 | 1.1093 | 0.2403 | 2.4748 | 3.9649 | 0.6580 |
| 3 | ID083 | 1.0343 | 0.9019 | 3.9468 | 2.7517 | 3.8612 | 4.1739 | 4.5200 |
| 3 | ID084 | 0.3210 | 0.4745 | 1.3036 | 0.8201 | 13.3174 | 8.9327 | 0.3123 |
| 3 | ID085 | 0.2792 | 0.1479 | 0.6656 | 0.3189 | 1.5816 | 0.4736 | 0.0609 |
| 3 | ID086 | 0.4105 | 0.3862 | 0.6707 | 0.8762 | 1.7924 | 0.7971 | 0.3084 |
| 3 | ID087 | 0.3197 | 1.3257 | 1.1204 | 0.5644 | 6.8267 | 2.3277 | 0.1604 |
| 3 | ID088 | 0.3427 | 0.7413 | 1.3561 | 0.7236 | 4.0882 | 2.3520 | 0.8146 |
| 3 | ID089 | 0.2572 | 0.0945 | 1.6880 | 2.7792 | 0.6416 | 0.2661 | 0.1143 |
| 3 | ID090 | 1.7491 | 2.7457 | 2.4999 | 3.6146 | 8.5127 | 7.4789 | 1.9698 |
| 3 | ID091 | 1.1746 | 1.2556 | 0.8704 | 0.4725 | 3.2676 | 0.8542 | 0.2450 |
| 3 | ID092 | 0.9663 | 0.8606 | 0.5114 | 8.1960 | 2.5100 | 2.8144 | 1.6811 |
| 3 | ID093 | 0.3389 | 0.1311 | 1.3055 | 2.2152 | 3.4183 | 2.2274 | 0.0589 |
| 3 | ID094 | 0.1822 | 0.3389 | 1.1886 | 0.4827 | 2.3103 | 1.1966 | 3.0352 |
| 3 | ID095 | 0.6807 | 0.4032 | 0.7248 | 0.3987 | 2.2314 | 2.4981 | 0.7710 |
| 3 | ID096 | 0.1865 | 0.2685 | 0.2743 | 0.4795 | 1.0456 | 0.6915 | 1.0843 |
| 3 | ID097 | 0.1213 | 0.1707 | 0.5239 | 1.3099 | 0.4416 | 2.2272 | 0.0316 |
| 3 | ID098 | 0.1893 | 0.2687 | 2.9401 | 1.6923 | 1.0328 | 0.7485 | 0.1323 |
| 3 | ID099 | 0.1566 | 0.3495 | 0.4938 | 4.9461 | 1.3176 | 0.8071 | 0.4642 |
| 3 | ID100 | 0.1422 | 0.3637 | 0.2808 | 2.0779 | 0.9510 | 0.4629 | 0.2882 |
| 3 | ID101 | 0.1637 | 0.4963 | 0.3230 | 0.6920 | 1.9816 | 2.0614 | 0.2092 |
| 3 | ID102 | 0.1563 | 0.3044 | 0.3087 | 2.2631 | 1.3015 | 0.3625 | 0.1434 |
| 3 | ID103 | 0.0361 | 0.1229 | 0.0716 | 0.9398 | 0.4958 | 0.2083 | 0.0314 |
| 3 | ID104 | 0.0962 | 0.5800 | 0.1174 | 1.3300 | 1.1529 | 0.2193 | 0.0559 |
| 3 | ID105 | 0.0647 | 0.5700 | 0.2110 | 5.5300 | 0.3600 | 0.4539 | 0.2218 |
| 3 | ID106 | 0.1164 | 2.9400 | 0.1848 | 0.0900 | 0.4233 | 0.3662 | 0.0758 |
| 3 | ID107 | 0.0320 | 0.1200 | 0.2550 | 2.5300 | 0.1052 | 0.1117 | 0.1686 |
| 3 | ID108 | 0.7400 | 0.6400 | 1.1489 | 1.0700 | 3.9633 | 2.8832 | 1.7059 |
| 3 | ID109 | 0.6213 | 1.9400 | 0.7478 | 5.3300 | 5.3395 | 3.6205 | 0.8559 |
| 3 | ID110 | 0.0902 | 0.2300 | 0.4794 | 0.7900 | 0.2443 | 0.3814 | 0.0901 |
| 3 | ID111 | 0.1519 | 0.7200 | 0.3372 | 1.1400 | 1.1728 | 0.7204 | 0.0925 |
| 3 | ID112 | 0.4096 | 17.7600 | 4.9724 | 4.9300 | 18.8909 | 33.9813 | 1.8609 |
| 3 | ID113 | 0.2819 | 0.5800 | 0.4771 | 0.6300 | 0.8225 | 0.9568 | 1.2587 |
| 3 | ID114 | 0.0355 | 2.5400 | 0.2968 | 1.4600 | 1.0126 | 1.3872 | 0.5260 |
| 3 | ID115 | 0.1493 | 1.4500 | 1.2183 | 0.7000 | 2.1790 | 1.2724 | 1.9272 |
| 3 | ID116 | 0.4565 | 1.4500 | 2.8159 | 7.5100 | 9.4101 | 7.7992 | 1.7858 |
| 3 | ID117 | 0.5712 | 0.5500 | 1.5874 | 3.6400 | 5.0332 | 2.9001 | 0.5146 |
| 3 | ID118 | 1.3877 | 4.1400 | 1.4625 | 4.0000 | 9.7924 | 1.3783 | 1.0827 |
| 3 | ID119 | 0.1966 | 0.4600 | 0.9649 | 3.1700 | 1.0981 | 1.9516 | 0.9537 |
| 3 | ID120 | 0.2682 | 0.2800 | 0.5281 | 6.4300 | 3.5687 | 1.8251 | 0.1774 |
| 3 | ID121 | 0.3090 | 1.1800 | 0.7511 | 4.9300 | 2.6442 | 2.6242 | 0.4219 |
| 3 | ID122 | 0.3720 | 0.3500 | 1.6150 | 0.7400 | 7.5641 | 1.5443 | 2.8169 |
| 4 | ID123 | 0.7595 | 0.2555 | 0.6401 | 26.4304 | 3.6947 | 0.9541 | 0.8052 |
| 4 | ID124 | 0.7110 | 1.1745 | 3.7892 | 1.7635 | 17.7841 | 7.7760 | 5.4650 |
| 4 | ID125 | 0.4156 | 2.4005 | 0.6329 | 1.0771 | 4.5975 | 1.5398 | 0.7267 |
| 4 | ID126 | 0.6870 | 1.0612 | 0.9329 | 0.4361 | 4.5096 | 2.9258 | 3.8704 |
| 4 | ID127 | 0.4917 | 0.7230 | 0.9712 | 4.7421 | 2.8848 | 3.1628 | 0.5510 |
| 4 | ID128 | 1.0156 | 1.6279 | 1.4275 | 3.0667 | 7.1977 | 3.2366 | 3.2662 |
| 4 | ID129 | 0.4723 | 3.1336 | 1.4937 | 2.5262 | 4.3149 | 1.6657 | 1.9947 |
| 4 | ID130 | 0.3726 | 0.2191 | 0.6375 | 2.5621 | 0.8889 | 0.7985 | 0.4111 |
| 4 | ID131 | 0.7245 | 0.4968 | 0.9869 | 4.8519 | 1.3472 | 1.3973 | 0.6968 |
| 4 | ID132 | 0.3380 | 0.2733 | 0.5068 | 1.4577 | 2.8848 | 3.6487 | 0.4564 |
| 4 | ID133 | 0.2840 | 0.4031 | 0.7916 | 0.8664 | 1.8079 | 1.5362 | 0.7107 |
| 4 | ID134 | 0.2805 | 0.3469 | 2.1972 | 1.9301 | 3.2112 | 1.2636 | 1.7144 |
| 4 | ID135 | 0.1853 | 0.2512 | 0.2075 | 0.3865 | 0.6531 | 0.5257 | 0.2504 |
| 4 | ID136 | 0.4254 | 0.3590 | 0.4992 | 0.6868 | 1.4343 | 0.9030 | 0.5753 |
| 4 | ID137 | 0.3378 | 0.1097 | 0.9005 | 0.9192 | 1.0519 | 0.8465 | 0.4188 |
| 4 | ID138 | 1.0528 | 3.0906 | 1.0474 | 2.6143 | 3.1789 | 2.2449 | 1.7898 |
| 4 | ID139 | 0.4316 | 17.5056 | 0.9129 | 3.9994 | 3.3029 | 2.8312 | 0.9214 |
| 4 | ID140 | 0.3962 | 18.0843 | 11.2401 | 0.7540 | 6.0586 | 0.4458 | 1.6230 |
| 4 | ID141 | 0.1816 | 4.8238 | 0.3354 | 0.3463 | 1.4803 | 3.1226 | 0.9211 |
