# Supplementary material for: Naturally acquired antibodies against 7 Streptococcus pneumoniae serotypes in Indigenous and non-Indigenous adults
Source: PLoS One. 2022 Apr 14;17(4):e0267051. doi: 10.1371/journal.pone.0267051 (PMC9009640; doi:10.1371/journal.pone.0267051)
Supplement: S3 Appendix — Indigenous adults southern Ontario (Group 1), Indigenous adults northwestern Ontario (Group 2), non-Indigenous adults Thunder Bay (Group 3), and non-Indigenous adults Kenora (Group 4). All data displayed are the original values. For our statistical analyses, the lower limit of detection was determined for each serotype according to the WHO pneumococcal ELISA protocol. The lower limits of detection are serotype 3 (0.035 μg/ mL), serotype 6B (0.088 μg/ mL), 9V (0.070 μg/ mL), 14 (0.124 μg/ mL), 19A (0.078 μg/ mL), 19F (0.130 μg/ mL) and 23F (0.031 μg/ mL). All values below the lower limits of detection were reported as half the value for statistical purposes. (DOCX) [file pone.0267051.s010.docx]

| Group | Participant ID | 3 | 6B | 9V | 14 | 19A | 19F | 23F |
| --- | --- | --- | --- | --- | --- | --- | --- | --- |
| 1 | ID001 | 0.6880 | 2.0816 | 0.8649 | 1.6503 | 3.5408 | 3.1905 | 0.4796 |
| 1 | ID002 | 0.0578 | 0.1205 | 0.0393 | 0.1373 | 0.2451 | 0.2566 | 0.0407 |
| 1 | ID003 | 0.2336 | 0.6779 | 0.4918 | 0.7313 | 1.0955 | 1.3449 | 0.1695 |
| 1 | ID004 | 0.3497 | 1.0421 | 1.0372 | 0.8480 | 1.5524 | 1.3258 | 0.2446 |
| 1 | ID005 | 0.1548 | 1.1485 | 0.1276 | 0.6264 | 0.7833 | 0.5132 | 0.1646 |
| 1 | ID006 | 0.1934 | 0.6379 | 0.1754 | 0.6489 | 0.7245 | 0.3651 | 0.1542 |
| 1 | ID007 | 0.3101 | 1.3355 | 0.4826 | 0.4800 | 1.3033 | 1.3978 | 0.2265 |
| 1 | ID008 | 1.0397 | 3.3885 | 1.1217 | 2.5127 | 4.7267 | 5.1413 | 0.8785 |
| 1 | ID009 | 0.6107 | 3.0187 | 0.6333 | 1.1794 | 4.4222 | 3.7478 | 0.7780 |
| 1 | ID010 | 1.1326 | 6.0485 | 2.8944 | 9.6632 | 7.4000 | 8.2500 | 1.2145 |
| 1 | ID011 | 0.5002 | 1.8015 | 0.5779 | 0.9160 | 1.6947 | 1.4724 | 0.3530 |
| 1 | ID012 | 0.7895 | 2.7842 | 0.6953 | 1.1057 | 4.1205 | 2.7398 | 0.6600 |
| 1 | ID013 | 0.2397 | 1.0975 | 0.2689 | 1.2839 | 1.1047 | 0.8356 | 0.2361 |
| 1 | ID014 | 0.1076 | 0.4937 | 0.0892 | 0.1584 | 0.5617 | 0.5166 | 0.1272 |
| 1 | ID015 | 0.3446 | 1.2139 | 0.5076 | 0.8070 | 1.4801 | 1.3180 | 0.2667 |
| 1 | ID016 | 0.7718 | 3.1771 | 0.6345 | 0.8825 | 3.7529 | 3.2832 | 0.6494 |
| 1 | ID017 | 0.3150 | 1.2576 | 0.3058 | 0.3312 | 1.1440 | 1.7046 | 0.2342 |
| 1 | ID018 | 0.0540 | 0.1559 | 0.0682 | 0.1830 | 0.2481 | 0.3022 | 0.0683 |
| 1 | ID019 | 0.2667 | 1.0494 | 0.5039 | 0.5144 | 1.5526 | 1.5654 | 0.3243 |
| 1 | ID020 | 0.4587 | 1.8923 | 0.4762 | 0.7245 | 2.6150 | 2.1895 | 0.5702 |
| 1 | ID021 | 0.1408 | 0.5152 | 0.3092 | 0.1148 | 0.6849 | 0.4680 | 0.1875 |
| 1 | ID022 | 0.2377 | 0.9942 | 0.2413 | 0.2519 | 1.0463 | 0.9686 | 0.2552 |
| 1 | ID023 | 0.2818 | 1.0030 | 0.2638 | 0.2169 | 1.4111 | 1.1698 | 0.3317 |
| 1 | ID024 | 0.1418 | 0.3474 | 0.1711 | 0.1888 | 0.6067 | 0.7368 | 0.1506 |
| 1 | ID025 | 0.4739 | 0.8433 | 0.8297 | 0.3480 | 1.4753 | 2.2629 | 0.2456 |
| 1 | ID026 | 0.3776 | 1.4781 | 0.9836 | 0.9682 | 2.1385 | 2.5833 | 0.3312 |
| 1 | ID027 | 0.1121 | 0.3978 | 0.2561 | 0.3519 | 0.7508 | 0.6139 | 0.0589 |
| 1 | ID028 | 0.1644 | 0.5140 | 0.1654 | 0.3543 | 0.6712 | 0.8539 | 0.1000 |
| 1 | ID029 | 0.2105 | 1.1031 | 0.2660 | 0.4012 | 1.0851 | 2.2699 | 0.1784 |
| 1 | ID030 | 0.7175 | 1.6095 | 1.1688 | 1.2651 | 3.3568 | 3.9702 | 0.3213 |
| 2 | ID031 | 0.1939 | 0.5774 | 0.2713 | 0.3265 | 0.7868 | 0.8144 | 0.1189 |
| 2 | ID032 | 0.4034 | 2.1732 | 0.1577 | 0.2192 | 2.4750 | 1.8318 | 0.4391 |
| 2 | ID033 | 0.2901 | 1.2954 | 0.3476 | 0.5803 | 1.7608 | 1.5483 | 0.2358 |
| 2 | ID034 | 0.1534 | 0.6168 | 0.2499 | 0.2651 | 0.7681 | 0.8599 | 0.0940 |
| 2 | ID035 | 0.6108 | 1.4879 | 0.3554 | 1.2436 | 2.0038 | 2.7971 | 0.5946 |
| 2 | ID036 | 0.1913 | 0.5251 | 0.4888 | 1.2175 | 0.6654 | 1.3894 | 0.1222 |
| 2 | ID037 | 0.0930 | 0.1487 | 0.3079 | 0.5737 | 0.2485 | 0.2358 | 0.0573 |
| 2 | ID038 | 0.3905 | 1.9114 | 0.8509 | 1.7914 | 2.3554 | 1.8884 | 0.4346 |
| 2 | ID039 | 0.3467 | 1.9374 | 0.9897 | 0.5249 | 1.7541 | 1.3860 | 0.4305 |
| 2 | ID040 | 0.3473 | 1.2956 | 0.3942 | 0.4967 | 2.7915 | 1.3333 | 0.3108 |
| 2 | ID041 | 0.2222 | 0.7753 | 0.2960 | 0.5084 | 1.1535 | 1.1698 | 0.3430 |
| 2 | ID042 | 0.0461 | 0.4087 | 0.1210 | 0.1496 | 0.2947 | 0.3243 | 0.0663 |
| 2 | ID043 | 0.1404 | 0.4836 | 0.1577 | 0.5540 | 2.1785 | 0.8796 | 0.1496 |
| 2 | ID044 | 0.3028 | 1.5224 | 0.5166 | 0.7164 | 1.8367 | 1.3470 | 0.3338 |
| 2 | ID045 | 0.5138 | 1.7629 | 0.9127 | 1.4767 | 1.3631 | 1.1955 | 0.3381 |
| 2 | ID046 | 0.2798 | 1.3843 | 0.2526 | 0.5721 | 1.2827 | 1.1035 | 0.3637 |
| 2 | ID047 | 0.2106 | 0.9421 | 0.4520 | 0.9815 | 0.7427 | 1.4671 | 0.1849 |
| 2 | ID048 | 0.4527 | 2.1390 | 0.8592 | 0.6365 | 2.2180 | 1.5191 | 0.4580 |
| 2 | ID049 | 0.1153 | 0.2489 | 0.0838 | 0.1368 | 0.3514 | 0.3644 | 0.0550 |
| 2 | ID050 | 0.3968 | 3.0857 | 0.9233 | 1.5658 | 3.5902 | 3.3727 | 0.2613 |
| 2 | ID051 | 0.2842 | 1.6471 | 0.7396 | 0.6839 | 1.9513 | 1.7626 | 0.2623 |
| 2 | ID052 | 0.3508 | 1.3525 | 0.2943 | 0.5792 | 4.3981 | 1.9819 | 0.2598 |
| 2 | ID053 | 0.2603 | 1.1203 | 0.2410 | 0.3695 | 1.2022 | 1.3634 | 0.1409 |
| 2 | ID054 | 0.0759 | 0.5450 | 0.1188 | 0.2379 | 0.4093 | 0.5190 | 0.0622 |
| 2 | ID055 | 0.3542 | 1.3384 | 0.6772 | 0.6393 | 1.2077 | 1.6649 | 0.3334 |
| 2 | ID056 | 0.2604 | 0.9313 | 0.1782 | 0.4381 | 0.8409 | 0.9758 | 0.1815 |
| 2 | ID057 | 0.8938 | 6.1438 | 1.9131 | 4.2316 | 4.8493 | 5.2048 | 0.5262 |
| 2 | ID058 | 0.9937 | 3.0083 | 1.5863 | 1.2885 | 4.0502 | 4.8840 | 0.7357 |
| 2 | ID059 | 0.2679 | 0.8874 | 0.6925 | 0.4565 | 0.9944 | 1.4424 | 0.2401 |
| 2 | ID060 | 0.4304 | 1.2966 | 0.3567 | 0.9697 | 1.3516 | 1.6195 | 0.2300 |
| 2 | ID061 | 0.7013 | 2.0397 | 0.8311 | 1.0848 | 2.5004 | 1.9939 | 0.4283 |
| 2 | ID062 | 0.8557 | 1.3716 | 0.6958 | 1.2517 | 2.0000 | 1.9680 | 0.4837 |
| 2 | ID063 | 0.8902 | 2.5336 | 0.7765 | 1.8026 | 3.5518 | 5.3609 | 0.6971 |
| 2 | ID064 | 0.2273 | 0.6034 | 0.2011 | 0.6346 | 0.7055 | 0.6348 | 0.1728 |
| 2 | ID065 | 0.4932 | 0.9498 | 0.2804 | 0.4774 | 1.2612 | 1.5519 | 0.3008 |
| 2 | ID066 | 0.6142 | 1.3491 | 0.4722 | 0.8394 | 1.4820 | 1.4458 | 0.3848 |
| 2 | ID067 | 0.1507 | 0.5231 | 0.2580 | 0.2254 | 0.6015 | 1.5819 | 0.1028 |
| 2 | ID068 | 0.2794 | 1.4026 | 0.2437 | 0.4854 | 1.8155 | 1.5470 | 0.2264 |
| 2 | ID069 | 0.1537 | 0.8142 | 0.8038 | 0.6412 | 0.8427 | 1.4664 | 0.2170 |
| 2 | ID070 | 0.2092 | 1.5965 | 1.1329 | 0.4385 | 1.3478 | 1.5974 | 0.2032 |
| 2 | ID071 | 0.2186 | 3.6333 | 0.3228 | 1.1565 | 2.1969 | 1.2920 | 0.2990 |
| 2 | ID072 | 0.5904 | 2.6255 | 0.5786 | 0.6965 | 2.3088 | 3.0616 | 0.6808 |
| 2 | ID073 | 0.4773 | 2.4440 | 1.0077 | 1.2180 | 2.1334 | 2.6500 | 0.4426 |
| 2 | ID074 | 0.2170 | 0.8217 | 0.3187 | 0.4141 | 1.7028 | 1.2067 | 0.2231 |
| 2 | ID075 | 0.1137 | 0.5569 | 0.1805 | 0.6469 | 0.9206 | 1.2102 | 0.1606 |
| 2 | ID076 | 0.0845 | 0.2229 | 0.2476 | 0.2197 | 0.2775 | 0.3630 | 0.0686 |
| 2 | ID077 | 1.1201 | 3.1003 | 1.2781 | 1.9773 | 5.4468 | 5.1834 | 1.2195 |
| 3 | ID078 | 0.2639 | 1.0685 | 0.4616 | 0.5632 | 1.5030 | 2.6970 | 0.1868 |
| 3 | ID079 | 0.2775 | 0.8237 | 0.6659 | 1.6008 | 1.5308 | 1.3029 | 0.2201 |
| 3 | ID080 | 0.4841 | 2.1793 | 0.7779 | 1.3397 | 2.5595 | 2.8430 | 0.4932 |
| 3 | ID081 | 0.5018 | 2.7849 | 0.9943 | 1.0636 | 2.2872 | 3.4690 | 0.4623 |
| 3 | ID082 | 1.1965 | 2.6146 | 0.4499 | 1.9897 | 4.4654 | 4.7566 | 0.8361 |
| 3 | ID083 | 0.5582 | 1.1427 | 1.3367 | 0.9685 | 2.6126 | 2.2567 | 0.5007 |
| 3 | ID084 | 0.3735 | 1.6788 | 0.5612 | 0.6900 | 2.0024 | 3.7445 | 0.5636 |
| 3 | ID085 | 1.2095 | 3.5948 | 1.2484 | 1.1707 | 4.1776 | 6.3427 | 0.8486 |
| 3 | ID086 | 0.1725 | 0.6268 | 0.2057 | 0.4781 | 0.6868 | 0.8856 | 0.1787 |
| 3 | ID087 | 0.5758 | 2.2015 | 1.0443 | 1.2540 | 2.4901 | 3.6447 | 0.3891 |
| 3 | ID088 | 0.7476 | 2.9970 | 0.5207 | 1.2009 | 4.1848 | 4.8756 | 0.4780 |
| 3 | ID089 | 0.1804 | 0.6960 | 0.1584 | 0.4312 | 1.1700 | 1.5241 | 0.2245 |
| 3 | ID090 | 0.1516 | 0.4835 | 0.1393 | 0.1931 | 0.5461 | 0.8431 | 0.1210 |
| 3 | ID091 | 0.0919 | 0.2807 | 0.0695 | 0.1828 | 0.3203 | 0.4609 | 0.0685 |
| 3 | ID092 | 0.3109 | 1.1152 | 0.3119 | 0.6090 | 1.4399 | 2.4509 | 0.2799 |
| 3 | ID093 | 0.0982 | 0.2983 | 0.0671 | 0.1590 | 0.3811 | 0.4885 | 0.0719 |
| 3 | ID094 | 0.3640 | 1.4547 | 0.3645 | 0.9161 | 2.2906 | 2.1700 | 0.3449 |
| 3 | ID095 | 0.2149 | 0.8512 | 0.3741 | 0.3759 | 1.0417 | 1.1757 | 0.2121 |
| 3 | ID096 | 0.2922 | 0.8453 | 0.2921 | 1.3518 | 1.1023 | 1.3105 | 0.2647 |
| 3 | ID097 | 0.1869 | 0.2577 | 0.0798 | 0.4420 | 0.3262 | 0.4155 | 0.0770 |
| 3 | ID098 | 0.2790 | 1.4158 | 0.3024 | 1.4714 | 1.5502 | 1.4184 | 0.2713 |
| 3 | ID099 | 0.3118 | 1.2429 | 0.2755 | 0.9377 | 1.5545 | 1.7704 | 0.2795 |
| 3 | ID100 | 0.1626 | 0.3942 | 0.1049 | 0.5438 | 0.5824 | 0.8221 | 0.1053 |
| 3 | ID101 | 0.2128 | 0.9012 | 0.1439 | 0.8032 | 0.9073 | 1.0584 | 0.1887 |
| 3 | ID102 | 0.1011 | 0.2292 | 0.0776 | 0.2183 | 0.3036 | 0.3523 | 0.0625 |
| 3 | ID103 | 0.1143 | 0.4635 | 0.1027 | 0.1877 | 0.5846 | 0.6237 | 0.0984 |
| 3 | ID104 | 0.4456 | 1.8620 | 0.5634 | 0.7518 | 2.6400 | 1.8950 | 0.3638 |
| 3 | ID105 | 0.0833 | 0.3328 | 0.0868 | 0.1223 | 0.4494 | 0.3838 | 0.0818 |
| 3 | ID106 | 0.4016 | 1.5431 | 0.5736 | 0.3443 | 2.3812 | 2.1751 | 0.2791 |
| 3 | ID107 | 1.7935 | 6.1530 | 1.9157 | 3.0967 | 6.6729 | 8.0335 | 1.3744 |
| 3 | ID108 | 0.4703 | 1.6544 | 0.3059 | 0.6046 | 2.5853 | 2.2600 | 0.3082 |
| 3 | ID109 | 0.7394 | 2.9494 | 0.7336 | 0.8232 | 4.4612 | 3.4798 | 0.4399 |
| 3 | ID110 | 0.7661 | 1.7890 | 0.3954 | 0.7949 | 2.4733 | 2.4962 | 0.4232 |
| 3 | ID111 | 0.7562 | 1.9536 | 0.8095 | 0.7167 | 2.7292 | 4.2945 | 0.4736 |
| 3 | ID112 | 0.2216 | 0.4895 | 0.1911 | 0.3911 | 1.1035 | 0.9431 | 0.1386 |
| 3 | ID113 | 0.2833 | 0.5188 | 0.2059 | 0.4374 | 0.9515 | 1.1897 | 0.1351 |
| 3 | ID114 | 0.2762 | 0.7299 | 0.2637 | 0.4283 | 1.1333 | 1.1128 | 0.1934 |
| 3 | ID115 | 0.1502 | 2.3139 | 0.1862 | 0.3051 | 0.8004 | 0.5608 | 0.1325 |
| 3 | ID116 | 0.0900 | 0.3917 | 0.2035 | 0.5242 | 0.3542 | 0.5522 | 0.1466 |
| 3 | ID117 | 0.3205 | 1.1268 | 0.2101 | 0.5680 | 1.4947 | 1.3350 | 0.2561 |
| 3 | ID118 | 0.2340 | 0.8852 | 0.1420 | 0.3032 | 0.7150 | 0.9168 | 0.1559 |
| 3 | ID119 | 0.6167 | 1.4553 | 1.4803 | 0.9137 | 1.7560 | 2.1248 | 0.3422 |
| 3 | ID120 | 0.5945 | 1.4827 | 0.5648 | 0.8696 | 1.3804 | 1.2244 | 0.1502 |
| 3 | ID121 | 0.3071 | 1.4927 | 0.3897 | 2.6681 | 0.7337 | 1.4280 | 0.1594 |
| 3 | ID122 | 0.2609 | 0.8319 | 0.1105 | 0.2168 | 1.2110 | 0.5638 | 0.1208 |
| 4 | ID123 | 0.1234 | 0.3883 | 0.2086 | 0.7497 | 1.3156 | 1.1806 | 0.1540 |
| 4 | ID124 | 0.2111 | 0.7442 | 0.3096 | 0.5485 | 1.3268 | 1.4339 | 0.1913 |
| 4 | ID125 | 0.3085 | 2.0223 | 0.3143 | 0.5738 | 3.4658 | 3.0208 | 0.3604 |
| 4 | ID126 | 0.2755 | 1.0678 | 0.6661 | 0.7542 | 2.0879 | 2.4696 | 0.3979 |
| 4 | ID127 | 0.2128 | 0.4959 | 0.1154 | 0.3976 | 1.0344 | 0.7657 | 0.1193 |
| 4 | ID128 | 0.1988 | 1.5257 | 0.4184 | 0.4371 | 1.0913 | 1.3590 | 0.1606 |
| 4 | ID129 | 0.3509 | 1.3586 | 0.8199 | 0.4339 | 1.9490 | 2.2178 | 0.3610 |
| 4 | ID130 | 0.0942 | 0.4424 | 0.0798 | 0.3031 | 0.3657 | 0.6694 | 0.0877 |
| 4 | ID131 | 0.1298 | 0.4420 | 0.0563 | 0.1323 | 0.4167 | 0.6675 | 0.0713 |
| 4 | ID132 | 0.4040 | 2.0331 | 0.2053 | 0.4787 | 1.3181 | 1.6573 | 0.2643 |
| 4 | ID133 | 0.4283 | 2.0205 | 0.6137 | 0.5877 | 2.7567 | 3.5970 | 0.3939 |
| 4 | ID134 | 0.2084 | 0.5867 | 0.3907 | 0.2276 | 0.8474 | 1.4261 | 0.1147 |
| 4 | ID135 | 0.1013 | 0.3119 | 0.1027 | 0.0959 | 0.4095 | 0.4716 | 0.0866 |
| 4 | ID136 | 0.2607 | 1.2700 | 0.5568 | 0.4763 | 1.1194 | 1.3894 | 0.1587 |
| 4 | ID137 | 0.9032 | 2.4511 | 0.4662 | 0.5966 | 4.9640 | 8.0175 | 0.9227 |
| 4 | ID138 | 0.2662 | 0.8618 | 0.2700 | 0.6365 | 0.9233 | 1.2601 | 0.2177 |
| 4 | ID139 | 0.1911 | 0.6838 | 0.1377 | 0.6550 | 0.7156 | 1.1667 | 0.0753 |
| 4 | ID140 | 0.0798 | 0.3091 | 0.0537 | 0.1291 | 0.3520 | 0.3230 | 0.0687 |
| 4 | ID141 | 0.2551 | 0.7418 | 0.4227 | 0.3751 | 1.0538 | 1.2945 | 0.1373 |
