# Supplementary material for: Naturally acquired antibodies against 7 Streptococcus pneumoniae serotypes in Indigenous and non-Indigenous adults
Source: PLoS One. 2022 Apr 14;17(4):e0267051. doi: 10.1371/journal.pone.0267051 (PMC9009640; doi:10.1371/journal.pone.0267051)
Supplement: S4 Appendix — Indigenous adults southern Ontario (Group 1), Indigenous adults northwestern Ontario (Group 2), non-Indigenous adults Thunder Bay (Group 3), and non-Indigenous adults Kenora (Group 4). All data displayed are the original values. For our statistical analyses, the lower limit of detection was determined for each serotype according to the WHO pneumococcal ELISA protocol. The lower limits of detection are serotype 3 (0.052 μg/ mL), serotype 6B (0.025 μg/ mL), 9V (0.036 μg/ mL), 14 (0.053 μg/ mL), 19A (0.025 μg/ mL), 19F (0.022 μg/ mL) and 23F (0.029 μg/ mL). All values below the lower limits of detection were reported as half the value for statistical purposes. (DOCX) [file pone.0267051.s011.docx]

| Group | Participant ID | 3 | 6B | 9V | 14 | 19A | 19F | 23F |
| --- | --- | --- | --- | --- | --- | --- | --- | --- |
| 1 | ID001 | 0.0664 | 0.0717 | 0.3803 | 0.1059 | 0.4592 | 0.0854 | 0.1474 |
| 1 | ID002 | 0.0523 | 0.0507 | 0.1690 | 0.1174 | 0.0899 | 0.0329 | 0.0256 |
| 1 | ID003 | 0.2291 | 0.0903 | 0.1443 | 0.2434 | 0.1067 | 0.0617 | 0.1443 |
| 1 | ID004 | 0.1079 | 0.0808 | 0.1064 | 0.1666 | 0.0876 | 0.0305 | 0.0074 |
| 1 | ID005 | 0.4252 | 0.3973 | 0.2274 | 0.5840 | 1.6657 | 0.1427 | 0.1338 |
| 1 | ID006 | 4.4803 | 0.2966 | 1.6207 | 1.0179 | 0.6751 | 0.3121 | 0.2496 |
| 1 | ID007 | 0.1143 | 0.0860 | 0.4579 | 0.7569 | 0.2584 | 0.0888 | 0.0381 |
| 1 | ID008 | 0.1394 | 0.0859 | 0.1043 | 0.1813 | 0.3122 | 0.1995 | 0.0964 |
| 1 | ID009 | 0.3594 | 0.8566 | 0.2766 | 0.6240 | 0.6628 | 0.2753 | 0.4561 |
| 1 | ID010 | 0.5504 | 0.1129 | 0.1225 | 0.3232 | 1.8912 | 0.2449 | 0.3513 |
| 1 | ID011 | 0.7744 | 0.5670 | 1.7073 | 1.4400 | 2.7694 | 1.4156 | 0.4855 |
| 1 | ID012 | 0.7514 | 0.5000 | 0.7230 | 1.9689 | 0.6871 | 0.2424 | 1.1818 |
| 1 | ID013 | 0.3656 | 3.8014 | 2.4845 | 1.5036 | 1.6918 | 1.4493 | 0.9242 |
| 1 | ID014 | 0.2102 | 0.1195 | 0.1442 | 0.1124 | 0.1961 | 0.0863 | 0.1233 |
| 1 | ID015 | 0.5797 | 0.1944 | 0.1279 | 0.4660 | 0.2866 | 0.2835 | 0.1930 |
| 1 | ID016 | 0.2321 | 0.1714 | 0.1239 | 0.1636 | 0.3623 | 0.1801 | 1.0185 |
| 1 | ID017 | 0.1472 | 0.3273 | 0.2018 | 0.2449 | 0.3190 | 0.1021 | 0.2172 |
| 1 | ID018 | 0.0279 | 0.0249 | 0.0393 | 0.0578 | 0.0605 | 0.0290 | 0.0482 |
| 1 | ID019 | 0.2687 | 0.0718 | 0.1148 | 0.5643 | 0.3915 | 0.3456 | 0.1039 |
| 1 | ID020 | 0.3081 | 0.1270 | 0.2396 | 0.5177 | 0.7048 | 0.1339 | 0.1650 |
| 1 | ID021 | 0.0485 | 0.0859 | 0.3062 | 0.1606 | 0.5652 | 0.0583 | 0.0477 |
| 1 | ID022 | 0.2349 | 0.0886 | 0.1181 | 0.2563 | 0.1377 | 0.0772 | 0.0693 |
| 1 | ID023 | 0.1263 | 0.1267 | 0.3988 | 0.4694 | 0.2360 | 0.1204 | 0.2712 |
| 1 | ID024 | 0.0894 | 0.1357 | 0.4177 | 0.2765 | 0.2962 | 0.2022 | 0.2505 |
| 1 | ID025 | 0.7449 | 1.4448 | 0.2157 | 2.3482 | 0.2668 | 0.1056 | 0.1998 |
| 1 | ID026 | 0.6156 | 0.0750 | 0.1670 | 1.4646 | 0.2165 | 0.1562 | 0.1535 |
| 1 | ID027 | 0.1315 | 0.2028 | 0.1557 | 0.2588 | 3.4558 | 0.1260 | 0.0413 |
| 1 | ID028 | 0.0279 | 0.0578 | 0.0174 | 0.0364 | 0.0438 | 0.0213 | 0.0252 |
| 1 | ID029 | 0.4777 | 0.2933 | 1.1395 | 0.3592 | 2.1372 | 0.2382 | 0.8485 |
| 1 | ID030 | 0.5544 | 0.0940 | 0.1990 | 0.7588 | 0.7008 | 0.6316 | 0.0517 |
| 2 | ID031 | 0.6089 | 0.1909 | 0.2283 | 0.3840 | 1.0086 | 0.1420 | 0.1590 |
| 2 | ID032 | 0.3092 | 0.1363 | 0.4637 | 0.5366 | 1.1246 | 0.1247 | 0.1217 |
| 2 | ID033 | 0.4090 | 0.0725 | 0.1439 | 0.0473 | 0.6319 | 0.0875 | 0.0851 |
| 2 | ID034 | 0.0415 | 0.0518 | 0.0559 | 0.0281 | 0.0771 | 0.0268 | 0.0438 |
| 2 | ID035 | 0.2783 | 0.1827 | 0.1717 | 0.4697 | 0.5629 | 0.2729 | 0.1516 |
| 2 | ID036 | 0.1271 | 0.6655 | 0.2252 | 0.2439 | 0.3473 | 0.1602 | 0.0610 |
| 2 | ID037 | 0.1297 | 0.1423 | 0.1328 | 0.0861 | 0.2287 | 0.0959 | 0.4273 |
| 2 | ID038 | 0.2321 | 0.0800 | 0.2903 | 0.9612 | 0.1963 | 0.0933 | 0.1585 |
| 2 | ID039 | 0.2598 | 0.0950 | 0.2816 | 0.2183 | 0.2232 | 0.0866 | 0.0338 |
| 2 | ID040 | 0.4843 | 0.1574 | 0.3845 | 0.2647 | 0.1252 | 0.0508 | 0.2316 |
| 2 | ID041 | 0.0523 | 0.0514 | 0.0965 | 0.2789 | 0.0663 | 0.0425 | 0.0326 |
| 2 | ID042 | 0.9475 | 0.2502 | 0.6282 | 0.0443 | 0.1510 | 0.1121 | 0.0870 |
| 2 | ID043 | 0.6247 | 0.1194 | 0.1775 | 0.2474 | 0.2237 | 0.1196 | 0.1078 |
| 2 | ID044 | 0.3122 | 0.1184 | 0.3877 | 0.4150 | 0.2086 | 0.0716 | 0.0703 |
| 2 | ID045 | 2.9211 | 0.1434 | 0.3859 | 0.2261 | 0.3046 | 0.2043 | 0.0826 |
| 2 | ID046 | 0.5612 | 0.1117 | 0.0927 | 0.4738 | 0.5437 | 0.0794 | 0.0939 |
| 2 | ID047 | 0.0772 | 0.0742 | 0.3359 | 0.2141 | 0.0935 | 0.0920 | 0.0609 |
| 2 | ID048 | 0.3030 | 0.2405 | 0.1845 | 0.4798 | 1.2122 | 0.2009 | 0.1817 |
| 2 | ID049 | 0.0383 | 0.0477 | 0.1401 | 0.0280 | 0.1008 | 0.0641 | 0.0363 |
| 2 | ID050 | 1.0023 | 0.6006 | 0.1249 | 0.1808 | 0.7220 | 0.2075 | 0.1764 |
| 2 | ID051 | 0.7878 | 0.2736 | 1.1795 | 0.3852 | 0.4968 | 0.5981 | 0.1175 |
| 2 | ID052 | 0.1875 | 0.1089 | 0.0938 | 1.8618 | 0.1301 | 0.0808 | 0.0548 |
| 2 | ID053 | 0.5912 | 0.0743 | 0.4195 | 0.1436 | 0.2169 | 0.0721 | 0.1117 |
| 2 | ID054 | 0.0763 | 0.0920 | 0.3763 | 0.0809 | 0.6964 | 0.1545 | 0.2973 |
| 2 | ID055 | 0.2023 | 0.1533 | 0.1383 | 0.1394 | 0.3799 | 0.0481 | 0.0815 |
| 2 | ID056 | 0.3101 | 0.8183 | 0.1357 | 0.2099 | 0.0632 | 0.0370 | 0.2315 |
| 2 | ID057 | 0.3405 | 0.1853 | 0.3670 | 1.7319 | 0.8378 | 0.5102 | 0.3174 |
| 2 | ID058 | 0.1859 | 0.6998 | 0.6883 | 2.4411 | 0.5971 | 0.1350 | 0.1036 |
| 2 | ID059 | 0.5763 | 0.2514 | 0.6639 | 0.2231 | 0.4248 | 0.1338 | 0.2526 |
| 2 | ID060 | 0.1920 | 0.0784 | 0.1395 | 0.1037 | 0.1803 | 0.0548 | 0.0439 |
| 2 | ID061 | 0.1696 | 0.0630 | 0.2299 | 0.3614 | 0.1909 | 0.0610 | 0.0667 |
| 2 | ID062 | 2.4239 | 1.4518 | 0.4824 | 0.5625 | 0.6991 | 0.7049 | 0.9171 |
| 2 | ID063 | 0.2355 | 0.2335 | 0.3567 | 0.5911 | 0.5276 | 0.3871 | 0.2909 |
| 2 | ID064 | 0.5951 | 0.1754 | 0.1186 | 0.3094 | 1.6022 | 0.1165 | 0.0387 |
| 2 | ID065 | 0.4824 | 0.3835 | 0.1002 | 0.5212 | 0.1065 | 0.0773 | 0.1424 |
| 2 | ID066 | 0.1014 | 0.1827 | 0.2572 | 0.1554 | 0.0984 | 0.0854 | 0.1990 |
| 2 | ID067 | 0.0366 | 0.0230 | 0.0780 | 0.0234 | 0.0525 | 0.0413 | 0.0250 |
| 2 | ID068 | 1.0291 | 0.1529 | 0.2362 | 0.2573 | 0.3171 | 0.2768 | 0.0681 |
| 2 | ID069 | 0.4158 | 0.1277 | 0.2699 | 0.3067 | 1.4843 | 0.1788 | 0.3450 |
| 2 | ID070 | 0.1191 | 0.2301 | 0.3250 | 0.2906 | 0.2113 | 0.1044 | 0.3190 |
| 2 | ID071 | 0.1769 | 1.0273 | 0.3295 | 0.5552 | 0.2568 | 0.1601 | 0.2728 |
| 2 | ID072 | 0.2999 | 0.2066 | 0.0916 | 0.1044 | 0.1207 | 0.0342 | 0.1098 |
| 2 | ID073 | 0.2285 | 0.1164 | 0.1428 | 0.2755 | 0.2607 | 0.1022 | 0.0800 |
| 2 | ID074 | 0.6563 | 0.1701 | 1.4641 | 0.1496 | 1.3199 | 0.3499 | 0.2781 |
| 2 | ID075 | 0.3413 | 0.4288 | 0.3011 | 0.9078 | 0.5501 | 0.2159 | 0.1945 |
| 2 | ID076 | 0.1123 | 0.0847 | 0.1085 | 0.1320 | 0.1443 | 0.1346 | 0.1159 |
| 2 | ID077 | 0.1983 | 0.1054 | 0.1447 | 0.4285 | 0.3930 | 0.1290 | 0.1305 |
| 3 | ID078 | 0.1990 | 0.1398 | 0.0852 | 0.4294 | 0.1894 | 0.2919 | 0.1042 |
| 3 | ID079 | 0.1406 | 0.0501 | 0.1864 | 0.3336 | 0.1310 | 0.0606 | 0.1175 |
| 3 | ID080 | 0.1560 | 0.0733 | 0.0653 | 0.0317 | 0.1042 | 0.0558 | 0.0468 |
| 3 | ID081 | 0.3157 | 0.0691 | 0.1397 | 0.0639 | 0.1143 | 0.1206 | 0.0921 |
| 3 | ID082 | 0.3358 | 0.1931 | 0.1072 | 0.1297 | 0.2687 | 0.1900 | 0.1044 |
| 3 | ID083 | 0.1241 | 0.0767 | 0.3640 | 0.2672 | 0.0943 | 0.0804 | 0.0755 |
| 3 | ID084 | 0.2786 | 0.1970 | 0.4395 | 0.0703 | 0.6843 | 0.7410 | 0.0509 |
| 3 | ID085 | 0.1166 | 0.0757 | 0.0956 | 0.0880 | 0.1717 | 0.0579 | 0.0417 |
| 3 | ID086 | 0.2122 | 0.0360 | 0.0438 | 0.1156 | 0.0763 | 0.0399 | 0.0266 |
| 3 | ID087 | 0.1818 | 0.1507 | 0.1147 | 0.3756 | 1.6899 | 0.1217 | 0.0263 |
| 3 | ID088 | 0.2465 | 0.1607 | 0.1012 | 0.1321 | 0.2844 | 0.1867 | 0.1019 |
| 3 | ID089 | 0.0734 | 0.0443 | 0.1171 | 0.2312 | 0.0616 | 0.0424 | 0.0533 |
| 3 | ID090 | 0.3810 | 0.1715 | 0.1977 | 0.1762 | 0.2776 | 0.1579 | 0.1496 |
| 3 | ID091 | 0.2265 | 0.1061 | 0.1045 | 0.0765 | 0.5024 | 0.1495 | 0.0554 |
| 3 | ID092 | 0.4822 | 0.0896 | 0.0479 | 0.2695 | 0.2073 | 0.1893 | 0.1340 |
| 3 | ID093 | 0.1440 | 0.0439 | 0.0970 | 0.1551 | 0.6292 | 0.3581 | 0.0284 |
| 3 | ID094 | 0.0304 | 0.0199 | 0.0189 | 0.0131 | 0.0807 | 0.0286 | 0.0479 |
| 3 | ID095 | 0.2854 | 0.0964 | 0.1472 | 0.2551 | 0.2880 | 0.0769 | 0.0574 |
| 3 | ID096 | 0.2231 | 0.1584 | 0.1322 | 0.1688 | 0.1329 | 0.0905 | 0.2084 |
| 3 | ID097 | 0.0483 | 0.0236 | 0.0927 | 0.0471 | 0.0638 | 0.0250 | 0.0151 |
| 3 | ID098 | 0.1067 | 0.0907 | 0.1808 | 0.1217 | 0.1302 | 0.0542 | 0.0465 |
| 3 | ID099 | 0.1211 | 0.1808 | 0.0927 | 1.1148 | 0.1082 | 0.0408 | 0.0382 |
| 3 | ID100 | 0.0921 | 0.0411 | 0.0945 | 0.2416 | 0.0831 | 0.0392 | 0.0253 |
| 3 | ID101 | 0.2903 | 0.1359 | 0.0744 | 0.1826 | 0.2469 | 0.1132 | 0.0807 |
| 3 | ID102 | 0.1357 | 0.0473 | 0.1023 | 0.0690 | 0.1179 | 0.0494 | 0.0303 |
| 3 | ID103 | 0.1186 | 0.0317 | 0.0238 | 0.0683 | 0.2509 | 0.0417 | 0.0194 |
| 3 | ID104 | 0.5233 | 0.2466 | 0.2307 | 0.6072 | 1.0156 | 0.1915 | 0.1775 |
| 3 | ID105 | 0.0397 | 0.0247 | 0.0673 | 0.1447 | 0.2511 | 0.0156 | 0.0147 |
| 3 | ID106 | 0.1544 | 0.0764 | 0.1076 | 0.0374 | 0.1812 | 0.0884 | 0.0294 |
| 3 | ID107 | 0.3139 | 0.1375 | 0.1382 | 0.6355 | 0.2356 | 0.1820 | 0.1185 |
| 3 | ID108 | 0.4859 | 0.2219 | 0.2245 | 0.2626 | 0.2314 | 0.1210 | 0.0910 |
| 3 | ID109 | 0.4019 | 0.3200 | 0.1262 | 0.5098 | 0.3496 | 0.1256 | 0.1188 |
| 3 | ID110 | 0.1882 | 0.0939 | 0.1685 | 0.0519 | 0.1188 | 0.0685 | 0.0922 |
| 3 | ID111 | 0.0741 | 0.2582 | 0.0623 | 0.0691 | 0.0751 | 0.0382 | 0.0235 |
| 3 | ID112 | 0.2366 | 0.2469 | 0.5350 | 0.1901 | 3.0563 | 0.6271 | 0.1291 |
| 3 | ID113 | 0.2245 | 0.3935 | 0.1108 | 0.0973 | 0.1403 | 0.1094 | 0.1374 |
| 3 | ID114 | 0.1040 | 0.0936 | 0.1356 | 0.3671 | 0.1906 | 0.1165 | 0.1232 |
| 3 | ID115 | 0.1150 | 0.1976 | 0.2725 | 0.0903 | 0.2739 | 0.0919 | 0.0525 |
| 3 | ID116 | 0.1930 | 0.4393 | 0.1513 | 0.2683 | 0.2829 | 0.1991 | 0.1954 |
| 3 | ID117 | 0.0309 | 0.0333 | 0.0350 | 0.1794 | 0.1024 | 0.0499 | 0.0128 |
| 3 | ID118 | 0.3264 | 0.2362 | 0.1340 | 0.2322 | 1.5543 | 0.0713 | 0.1245 |
| 3 | ID119 | 0.2975 | 0.0869 | 0.2897 | 0.2315 | 0.2791 | 0.2053 | 0.0489 |
| 3 | ID120 | 0.0852 | 0.0475 | 0.2093 | 0.4183 | 0.1725 | 0.0734 | 0.3005 |
| 3 | ID121 | 0.3941 | 0.1539 | 0.1009 | 0.4499 | 0.1421 | 0.0828 | 0.0464 |
| 3 | ID122 | 0.0826 | 0.0650 | 0.1288 | 0.0999 | 0.6045 | 0.1001 | 0.1760 |
| 4 | ID123 | 0.2835 | 0.1639 | 0.2217 | 1.2784 | 0.7478 | 0.1648 | 0.2161 |
| 4 | ID124 | 0.3928 | 0.3059 | 1.9823 | 0.0655 | 0.8117 | 0.0954 | 0.5994 |
| 4 | ID125 | 0.1182 | 0.0813 | 0.0299 | 0.0361 | 0.1705 | 0.0578 | 0.0498 |
| 4 | ID126 | 0.1634 | 0.0615 | 0.0493 | 0.0539 | 0.0968 | 0.0406 | 0.0615 |
| 4 | ID127 | 0.4724 | 0.0766 | 0.0866 | 0.5419 | 0.1636 | 0.1399 | 0.0378 |
| 4 | ID128 | 0.3373 | 0.1902 | 0.4833 | 0.1292 | 0.2330 | 0.1362 | 0.0722 |
| 4 | ID129 | 0.1960 | 0.1378 | 0.2784 | 0.1586 | 0.2668 | 0.0916 | 0.1574 |
| 4 | ID130 | 0.0784 | 0.0639 | 0.0588 | 0.1022 | 0.0455 | 0.0228 | 0.0228 |
| 4 | ID131 | 0.0723 | 0.0456 | 0.0848 | 0.1866 | 0.0721 | 0.0442 | 0.0360 |
| 4 | ID132 | 0.1513 | 0.0557 | 0.0515 | 0.0713 | 0.1892 | 0.1290 | 0.0498 |
| 4 | ID133 | 0.1429 | 0.0998 | 0.1107 | 0.0806 | 0.1462 | 0.0659 | 0.0423 |
| 4 | ID134 | 0.0371 | 0.0286 | 0.0866 | 0.0550 | 0.1560 | 0.0411 | 0.1140 |
| 4 | ID135 | 0.0664 | 0.0165 | 0.0142 | 0.0188 | 0.0601 | 0.0350 | 0.0141 |
| 4 | ID136 | 0.6070 | 0.1141 | 0.1646 | 0.1497 | 0.1466 | 0.0843 | 0.2181 |
| 4 | ID137 | 0.2202 | 0.0780 | 0.0627 | 0.0808 | 0.1415 | 0.1035 | 0.0650 |
| 4 | ID138 | 0.2722 | 0.1948 | 0.1169 | 0.7226 | 0.2154 | 0.1199 | 0.1312 |
| 4 | ID139 | 0.0960 | 0.4375 | 0.0511 | 0.6002 | 0.0900 | 0.0538 | 0.0571 |
| 4 | ID140 | 0.7690 | 0.3417 | 0.9379 | 0.7919 | 0.5570 | 0.3092 | 0.1802 |
| 4 | ID141 | 0.2605 | 0.0585 | 0.0575 | 0.0709 | 0.2619 | 0.1066 | 0.0778 |
