# Supplementary material for: Naturally acquired antibodies against 7 Streptococcus pneumoniae serotypes in Indigenous and non-Indigenous adults
Source: PLoS One. 2022 Apr 14;17(4):e0267051. doi: 10.1371/journal.pone.0267051 (PMC9009640; doi:10.1371/journal.pone.0267051)
Supplement: S5 Appendix — (DOCX) [file pone.0267051.s012.docx]

Inclusivity in global research

PLOS’ policy on inclusivity in global research aims to improve transparency in the reporting of research performed outside of researchers’ own country or community and ensures that PLOS publications reporting global research adhere to high standards for research ethics and authorship. Authors of relevant research articles may be asked to complete the questionnaire below, which outlines ethical, cultural, and scientific considerations specific to inclusivity in global research. This questionnaire may be requested when researchers have travelled to a different country to conduct research, if research uses samples collected in another country, research with Indigenous populations or their lands, or if research is on cultural artefacts. Researchers travelling to another country solely to use laboratory equipment will not normally be required to complete the questionnaire. However, the questionnaire can be requested at the journal’s discretion for any submission – if you have been requested to complete this questionnaire by the PLOS journal you submitted to, please do so.

Please complete the questionnaire below and include this as a Supporting Information file with your manuscript. Note that if your paper is accepted for publication, this checklist will be published with your article in the supporting information files. Please ensure that you reference the checklist in the main body of your manuscript. We suggest adding a subsection ‘Inclusivity in global research’ to your Methods section and adding the following sentence: “Additional information regarding the ethical, cultural, and scientific considerations specific to inclusivity in global research is included in the Supporting Information (SX Checklist)”

The questions have been designed to be applicable to a wide range of study types, and there are subsections for both human subjects research and non-human subjects research. If any of the questions are not relevant to your research please mark them as “N/A” as appropriate.

**Ethical considerations, permits and authorship**

*This section is applicable to all research types.*

Provide details as to who granted permissions and/or consent for the study to take place in the Methods section of your manuscript. This should include the names of **all** ethics boards, governmental organizations, community leaders or other bodies that provided approval for the study. If individuals provided approval refer to these people by their role or title but do not list their name(s).

Reported on page number: 6

If there were any deviations from the study protocol after approval was obtained please provide details of these changes in the Methods section of your manuscript.
Did this study involve local collaborators that are residents of the country where the research was conducted or members of the community studied? If you do not have any authors from said communities, please provide an explanation for this below.

N/A

Reported on page number:

Response: Everyone listed as an author met PLOS’ criteria for authorship as indicated below.

“The ICMJE lists four conditions for authorship credit. Authors must meet all four conditions in order to be listed.

- Substantial contributions to conception and design, acquisition of data, or analysis and interpretation of data, and
- Drafting the article or revising it critically for important intellectual content, and
- Final approval of the version to be published, and
- Agreement to be accountable for all aspects of the work in ensuring that questions related to the accuracy or integrity of any part of the work are appropriately investigated and resolved.”

Everyone listed as an author should meet PLOS’ criteria for authorship and all individuals who meet these criteria should be included in the author byline, rather than the acknowledgements. Authorship criteria is based on the International Committee of Medical Journal Editors (ICMJE) Uniform Requirements for Manuscripts Submitted to Biomedical Journals - for further information please see here: <https://journals.plos.org/plosone/s/authorship>.

**Human subjects research (e.g. health research, medical research, cross-cultural psychology)**

Did you obtain written informed consent from a representative of the local community or region before the research took place? How did you establish who speaks for the community? Details of written informed consent obtained from study participants should be reported separately in the Methods section of your manuscript.

**Outline of the engagement process with Aboriginal peoples**

This document is based on the experience of Dr. Ulanova’s research group (Northern Ontario School of Medicine) acquired while working on the project “Immunological basis for increased burden of invasive bacterial disease among First Nations in Northern Ontario” since 2012.

In designing and conducting this research we have fully adhered to the core principles of Ownership Control Access and Possession as defined by the National Aboriginal Health Organization and the Tri-Council Policy Statement “Ethical Conduct for Research Involving Humans” (TCPS2), specifically those outlined in Chapter 9 “ Research Involving the First Nations, Inuit and Métis Peoples of Canada.”

**Essential steps in the engagement process**

1. Raising awareness and obtaining support from Aboriginal organizations
2. Using the media to communicate the research question

*We have found that a press release through a public organization (e.g., university or hospital) outlining the problem and the way our research can help to address it often results in interest from other media outlets including Aboriginal ones. Local and specifically Aboriginal newspapers can be approached and they may accept submissions raising public awareness of the proposed research.*

1. Taking opportunities to present the research plan to Aboriginal organizations and asking them for input regarding project design and approach

1. Requesting letters of endorsement from Aboriginal communities and organizations

*Prior to starting the project we engaged in extensive consultation with a variety of stakeholders.  Following review of our research methodology we received letters of endorsement from Nishnawbe Aski Nation (political territorial organization representing 49 First Nation communities within Northern Ontario), The Metis Nation of Ontario, Red Rock Indian Band (Lake Helen First Nation), Bingwi Neyaashi Anishinaabek (Sand Point First Nation), and Fort William First Nation.* *This was very helpful with regards to establishing research partnerships with individual communities.*

1. Consulting with Aboriginal Elders to ensure that the proposed research is culturally appropriate

*Consultations with the Lakehead University Ogimaawin-Aboriginal Elders Council and individual Elders in the region were very helpful.*

1. The process of community engagement
2. Approaching the councillor with the health portfolio or the health director in the Aboriginal community: sending project information in a short and straight forward format (e.g., bullet point brochure) and including letters of support from others

*We have found that initial contact via e-mail with following up by phone a week later is the most productive approach.*

1. Asking for an opportunity to present the proposal during a council meeting
2. Asking for input from the community leadership regarding research design, methodology, and overall approach

3. Continuing collaboration/partnership with Aboriginal community

a) Establishing a Community Research Assistant (CRA) Position to increase awareness of the research project and facilitate participation of individual community members

*We have hired two CRAs in 2 First Nations communities who greatly helped to advance the project, via choosing the most efficient methods of community members’ engagement in research including posters, community gatherings, or approaching members individually. It is essential to provide paid training for CRAs to ensure they are prepared to answer any questions or concerns regarding all aspects of the research project.*

b) Regularly updating our partners through information sessions and progress reports as results are analysed; sharing research results with communities

c) Continuing raising awareness of the progress and the importance of the work through regional Aboriginal media

How did members of the local community provide input on the aims of the research investigation, its methodology, and its anticipated outcome(s)?

*We have hired two CRAs in 2 First Nations communities who greatly helped to advance the project, via choosing the most efficient methods of community members’ engagement in research including posters, community gatherings, or approaching members individually. It is essential to provide paid training for CRAs to ensure they are prepared to answer any questions or concerns regarding all aspects of the research project.*

When engaging with the local community, how did you ensure that the informed consent documents and other materials could be understood by local stakeholders?

Will the findings of the research be made available in an understandable format to stakeholders in the community where the study was conducted (e.g. via a presentation, summary report, copies of publications, etc.)? Please provide details of how this will be achieved.

Regularly updating our partners through information sessions and progress reports as results are analysed; sharing research results with communities.

**Non-human subjects research using specimens/ animals collected as part of the study, or those housed in archival collections. Examples include archaeology, paleontology, botany and zoology.**

Did the permission you obtained from a local authority to perform the study include an agreement on access to outputs and benefit sharing? This may include procedures to enable fair distribution of the benefits and resources arising from the research performed. Please include any details of Prior Informed Consent and Benefit Sharing Agreements obtained. These may be required by field-specific regulations, for example the Convention on Biological Diversity (CBD) and the associated Nagoya Protocol.

N/A

If the material used in your study was imported, please A) provide the year it was imported and B) indicate whether permits were obtained to import/export the materials used, C) provide details of any permits obtained. If this information is not available, please indicate this.

N/A

If you used archival specimens, please state how the material used in your study was acquired by the institute it is held in and provide details of any permits obtained for the original excavations/ sample collection. If this information is not available, please indicate this.

N/A

How was the potential cultural significance of the materials collected in your study to local communities considered in your research design? Were Indigenous peoples and/or local researchers and institutions involved with archaeological excavations / collection of specimens? If so, please provide a description of their involvement.

N/A

If your manuscript includes photographs of human remains please indicate whether authors obtained permission from descendants or affiliated cultural communities to do so.

N/A
